# Medial temporal lobe tau–neurodegeneration mismatch from structural imaging and plasma biomarkers

**DOI:** 10.1093/brain/awag105

**Published:** 2026-04-28

**Authors:** Xueying Lyu, Nidhi S Mundada, Christopher A Brown, Niyousha Sadeghpour, Emily McGrew, Long Xie, Yue Li, Anika Wuestefeld, Michael Tran Duong, Philip Cook, James Gee, Ilya M Nasrallah, Alice S Chen-Plotkin, Leslie M Shaw, Dawn Mechanic-Hamilton, Laura E M Wisse, Paul A Yushkevich, Sandhitsu R Das, David A Wolk

**Affiliations:** Department of Bioengineering, University of Pennsylvania, Philadelphia, PA 19104, USA; Department of Bioengineering, University of Pennsylvania, Philadelphia, PA 19104, USA; Department of Neurology, University of Pennsylvania, Philadelphia, PA 19104, USA; Department of Radiology, University of Pennsylvania, Philadelphia, PA 19104, USA; Department of Radiology, University of Pennsylvania, Philadelphia, PA 19104, USA; Department of Radiology, University of Pennsylvania, Philadelphia, PA 19104, USA; Department of Radiology, University of Pennsylvania, Philadelphia, PA 19104, USA; Department of Clinical Sciences Lund, Lund University, Lund SE-221 00, Sweden; Department of Radiology, University of Pennsylvania, Philadelphia, PA 19104, USA; Department of Radiology, University of Pennsylvania, Philadelphia, PA 19104, USA; Department of Radiology, University of Pennsylvania, Philadelphia, PA 19104, USA; Department of Radiology, University of Pennsylvania, Philadelphia, PA 19104, USA; Department of Neurology, University of Pennsylvania, Philadelphia, PA 19104, USA; Department of Bioengineering, University of Pennsylvania, Philadelphia, PA 19104, USA; Department of Neurology, University of Pennsylvania, Philadelphia, PA 19104, USA; Department of Clinical Sciences Lund, Lund University, Lund SE-221 00, Sweden; Department of Radiology, University of Pennsylvania, Philadelphia, PA 19104, USA; Department of Neurology, University of Pennsylvania, Philadelphia, PA 19104, USA; Department of Neurology, University of Pennsylvania, Philadelphia, PA 19104, USA

**Keywords:** Alzheimer’s disease, medial temporal lobe, LATE-NC, resilience, plasma ptau_217_, mismatch

## Abstract

Although tau pathology is closely associated with neurodegeneration in Alzheimer’s disease, our prior work using multi-modality imaging revealed that mismatch between tau (T) and neurodegeneration (N) might reflect contributions from non-Alzheimer’s disease processes. The medial temporal lobe (MTL), an early site of Alzheimer’s disease pathology, is also a common target of co-pathologies, such as limbic-predominant age-related TDP-43 encephalopathy neuropathological change (LATE-NC), often following an anterior-to-posterior atrophy gradient. Given the susceptibility of MTL to co-pathologies, here we explored T-N mismatch specifically within MTL using plasma ptau_217_ and MTL morphometry for identifying vulnerabilities and resilience in cognitively impaired or unimpaired patients with Alzheimer’s disease.

We parcellated the MTL into 100 spatially contiguous segments and calculated their T-N mismatch using plasma ptau_217_ as a measure for T and thickness as a marker of N. Based on these mismatch profiles, we clustered 447 amyloid-positive individuals from the Alzheimer’s Disease Neuroimaging Initiative cohort into data-driven T-N phenotypes. We characterized the T-N phenotypes by examining their cross-sectional and longitudinal atrophy both within the MTL and across the whole brain, in addition to cognitive trajectories. This framework was replicated in an independent cohort and, finally, translated to a real-world clinical sample of 50 patients undergoing anti-amyloid therapy.

Clustering identified three T-N phenotypes with different MTL T-N mismatch profiles, atrophy patterns and cognitive outcomes, despite comparable Alzheimer’s disease severity. The ‘canonical’ group, characterized by low T-N residuals (N ∼ T), showed Alzheimer’s disease-like neurodegeneration patterns. The ‘vulnerable’ group, characterized by disproportionately greater neurodegeneration than tau (N > T), showed atrophy primarily in the anterior MTL that extended into temporal-limbic regions, in both cross-sectional and longitudinal analyses. This group also exhibited neurodegeneration that preceded estimated tau onset and experienced faster cognitive decline across multiple domains, aligning with the typical characteristics of mixed LATE-NC with Alzheimer’s disease. In contrast, the ‘resilient’ group (N < T) showed minimal atrophy and preserved cognitive function. These phenotypes were reproducible in an independent research cohort. Importantly, in a feasibility study applying the model developed from the Alzheimer’s Disease Neuroimaging Initiative to a clinical cohort of patients receiving lecanemab, we identified vulnerable individuals with LATE-like atrophy patterns. This highlights its potential utility for identifying individuals with co-pathology in clinical settings.

Our findings demonstrate that T-N mismatch within MTL using MRI and plasma biomarkers can reveal Alzheimer’s disease groups with varying vulnerability/resilience, with the vulnerable group displaying structural and cognitive outcomes suggestive of LATE-NC. This approach offers a cost-effective strategy for clinical trial stratification and precision medicine for Alzheimer’s disease therapeutics.

## Teaser

Lyu *et al.* combined MRI-based medial temporal lobe morphometry with plasma ptau_217_ to assess tau-neurodegeneration mismatch in Alzheimer's disease, identifying three subgroups with distinct mismatch profiles, atrophy patterns, and cognitive trajectories despite similar disease severity.

## Introduction

The recent Alzheimer’s Association Workgroup revised diagnostic criteria built upon the foundation of the previously described ‘ATN framework’, which was a first attempt at a biological definition of Alzheimer’s disease (AD).^1,2^ Based on these criteria, amyloid-β plaques (A) and tau-based neurofibrillary tangles are the defining pathologies of AD^1,2^ that mark its presence and stage, respectively. However, AD is a heterogeneous condition commonly associated with additional pathologies, such as TAR DNA-binding protein 43 (TDP-43), α-synuclein and cerebrovascular disease. Detection of the presence of these co-pathologies and characterization of their role in disease progression are a critical knowledge gaps in the context of emergent disease-modifying therapies.^3,4^ The lack of specific and quantitative molecular biomarkers of these co-pathologies (e.g. TDP-43)^5-10^ is a major barrier to such characterization.

The medial temporal lobe (MTL)^11^ is a site of early neurodegeneration in AD. Most notably, tau (T) accumulation in the cerebral cortex typically begins in Brodmann area 35 (BA35) and the entorhinal cortex, then spreads to the hippocampus proper.^12-16^ The accumulation of T drives neurodegeneration (N) within the MTL, making it a clinically important region to aid in early AD diagnosis. Nevertheless, the N in MTL is not solely driven by T, because the region also serves as a critical locus of early pathology for co-pathologies in addition to AD. Limbic-predominant age-related TDP-43 encephalopathy neuropathological change (LATE-NC),^10,17^ which is present in ≤50% of patients with AD,^17^ also involves MTL structures, with involvement of the amygdala, then hippocampus and entorhinal cortex defining stage 1 and stage 2 of this condition, respectively.^18,19^ Moreover, previous work has found that there is an anterior-to-posterior gradient of involvement of LATE-NC along the long axis of the MTL, most prominently affecting anterior MTL regions and extending into anterior medial temporal polar regions.^18,20^ Additionally, other pathologies, such as α-synuclein and vascular disease, might also contribute to MTL structural changes, although they might be less specific for the MTL in terms of their downstream neurodegeneration.^18,21-23^ Therefore, characterization of the heterogeneity within the MTL is crucial for advancing a precision medicine approach in AD therapeutics.

Despite the close link between the burden of T and downstream N^12,24-27^ in AD, there is still considerable variability in this relationship across individuals. One potential source of this variability is the presence of co-pathologies that might have independent effects on measures of neurodegeneration. In our prior work,^28-30^ we quantified this variability by determining the degree of mismatch in the relationship between T and N across the whole brain using either MRI or ^18^F-fluorodeoxyglucose PET and tau PET as a surrogate of N and T, respectively. By examining maps of residuals between markers of T and N across the brain and using clustering techniques to find groups with characteristic T/N residual patterns, we identified distinct T-N mismatch phenotypes. Based on both *in vivo* and *ex vivo* studies,^28-30^ we found that these T-N phenotypes were enriched with specific co-pathologies (e.g. TDP-43 and vascular disease).

Given the susceptibility of MTL to multiple pathologies, particularly LATE-NC,^10,18,23^ in this work, we specifically investigated the T-N mismatch within this region by integrating structural MRI as a marker of N and a plasma biomarker as a marker of T. Plasma ptau_217_, a non-invasive yet highly specific AD biomarker, has been shown to have high sensitivity for the presence of cerebral amyloid-β as measured by amyloid-β PET, but also to be related to tau burden as measured by tau PET.^31-33^ Although tau PET might be more tightly linked to neurofibrillary tangle burden and provides spatial information, plasma ptau_217_ is a more practical and cost-effective measure for clinical practice. Given that MRI is also routinely acquired in clinical practice, especially in the course of anti-amyloid treatment, in the present analysis we set out to use plasma ptau_217_ as a measure of T and MTL thickness, combined with amygdala volume, as a surrogate of N. To study T-N mismatch at a finer spatial resolution, we divided the MTL into 100 segments of approximately equal size, referred to as ‘super-points’. This more granular representation was designed to capture spatial heterogeneity in T-N mismatch within the MTL better than using larger anatomically defined subregions (e.g. cornu ammonis 1, entorhinal cortex).

Using this simplified T-N framework, we first used a data-driven clustering approach to cluster amyloid-β-positive (A+) individuals with and without cognitive impairment from ADNI into a discrete set of groups based on similarity of T-N mismatch patterns across the 100 MTL super-points and amygdala. Based on our prior whole-brain T-N mismatch studies,^28-30^ here we anticipated identifying distinct T-N groups with negative residuals (N > T; vulnerable) or positive residuals (N < T; resilient) in MTL. Second, we examined cross-sectional and longitudinal atrophy trajectories in the MTL and across the whole brain, along with cognitive profiles, in these T-N groups. We hypothesized that the vulnerable group would be enriched in LATE and would exhibit LATE-like biomarker patterns, including an anterior-to-posterior gradient of atrophy in the MTL and a more impaired cognitive profile, predominantly in memory. Third, we further examined relationships of cognitive and neurodegenerative trajectories anchored to a time of tau positivity onset estimated using the sampled iterative local approximation (SILA) approach.^34^ Next, we replicated this approach in an independent cohort to assess its reproducibility. Finally, we applied the method to a real-world clinical cohort of patients treated with anti-amyloid-β therapy to evaluate its translational potential.

## Materials and methods

### Participants

Data used in the preparation of this article were obtained from the Alzheimer’s Disease Neuroimaging Initiative (ADNI) database (adni.loni.usc.edu), with description in the Supplementary material, ‘Participants’ section.

Participants were selected according to the criteria detailed in the Supplementary material, ‘Participants' section. We identified 469 A+ patients from ADNI with a pair of plasma ptau_217_ and T1-weighted MRI scan within 1 year apart, based on data accessed as of March 2025. After excluding 11 individuals owing to image processing failures (see the 'MTL thickness and subregional volume estimation' section) and another 11 for failed quality control for segmentation, a total of 447 A+ individuals were included in the analysis. The average time difference between the MRI scan and ptau_217_ measurement was 0.84 (±1.97) months. Detailed clinical characteristics of this cohort, including age, sex, Clinical Dementia Rating Sum of Boxes (CDRSB) and ptau_217_, are provided in Supplementary Table 1. We additionally included 123 amyloid-β-negative (A−) cognitively unimpaired individuals from ADNI as a control group. The average age of this group was 74.6 ± 6.7 years, comprising 51 females and 72 males.

To form an independent replication cohort, we included 108 A+ individuals from the University of Pennsylvania Alzheimer’s Disease Research Center (Penn ADRC), applying the same patient selection criteria as were used for ADNI. Data were collected from April 2016 to January 2025. The average interval between MRI and plasma ptau_217_ measurement was 3.6 ± 3.4 months. Clinical details for the Penn ADRC cohort are provided in Supplementary Table 1.

Finally, as a clinical translation cohort, we included 50 patients with MCI or mild dementia attributable to AD from the University of Pennsylvania Anti-Amyloid Therapy Monitoring (ATM) programme, all of whom have received lecanemab. All patients underwent MRI and plasma biomarker measurement prior to starting treatment, with data collected between May 2024 and January 2025. All participants provided informed consent in accordance with protocols approved by the University of Pennsylvania Institutional Review Board. The average interval between ptau_217_ and MRI in this group was 4.3 ± 2.4 months. Clinical details for ATM cohorts are also summarized in Supplementary Table 1.

### Image acquisition and processing

#### Image acquisition

T1-weighted MRI scans were required for ADNI, Penn ADRC and ATM cohorts. The image acquisition details for each cohort are outlined in the Supplementary material, ‘Image acquisition' section.

#### Determination of amyloid-β status

Amyloid-β status for ADNI participants was determined based on amyloid-β PET imaging, using tracer- and reference-specific standardized uptake value ratio (SUVR) thresholds by UC Berkeley. Given that most of participants from ADNI had amyloid PET, we used amyloid-β PET imaging for determination of amyloid status, which still remains the gold standard for amyloid classification. For the Penn ADRC cohort, amyloid-β status was based on visual reads of amyloid PET scans from a neuroradiologist with nuclear medicine training when available. For Penn ADRC participants without amyloid-β PET, amyloid-β status was determined using the ptau_217_:Aβ42 ratio, with a cut-off of >0.0055 indicating amyloid-β positivity. This threshold corresponds to 95% sensitivity and ∼90% overall classification accuracy against amyloid PET.^35^

#### MTL thickness and subregional volume estimation

MTL thickness was derived through a previously validated automatic pipeline.^36^ Initially, the MTL was segmented into subregions (anterior and posterior hippocampus, amygdala, entorhinal cortex, parahippocampal cortex, BA35 and BA36) from T1-weighted MRI using the Automated Segmentation of Hippocampal Subfields algorithm/software (ASHS),^37-39^ and volumes obtained. Intracranial volume was also estimated by ASHS. Next, an estimation of thickness was performed using the Cortical Reconstruction for ASHS pipeline (CRASHS).^36^ Detailed description of CRASHS has been outlined here.^36^ Briefly, it generates participant-specific grey/white, pial and mid-surfaces as triangular meshes for the MTL as the input for estimating thickness. To allow group-level morphometric analysis, individual MTL surfaces were aligned to a population template using diffeomorphic surface registration.

#### MTL super-points parcellation

We developed a super-point partitioning approach to parcellate the MTL further into 100 bilateral, spatially contiguous segments of approximately equal size, referred to as ‘super-points’, for cortical thickness analysis. This was done to allow more granular T-N mismatch analyses than using larger anatomically defined subregions. We defined the super-points once on CRASHS surface template, which is derived from inflated MTL mid-surfaces of a set of individuals on the AD continuum and represents a smooth ‘average’ MTL shape.^36^ Our prior work^28-30^ demonstrated stable clustering performance with 100 input features, and we therefore selected this number to balance spatial resolution with stability. The number of super-points assigned to each subregion was approximately proportional to its surface area, hence larger regions were represented by more partitions. Within each region, we constructed a triangle adjacency graph and applied PyMetis-based clustering^40^ to generate spatially contiguous super-points, ensuring that partitions remained confined within the anatomical boundaries of each region. Using the vertex-wise MTL thickness maps generated by CRASHS, we calculated the mean thickness for each super-point for each participant. This process produced MTL thickness estimates for 100 super-points.

#### Whole-brain cortical thickness processing

In addition to MTL thickness, T1-weighted MRI scans were processed using the ANTs cortical thickness pipeline,^41^ which includes intensity inhomogeneity correction and tissue segmentation. A multi-atlas segmentation approach^42^ was used to parcellate the scans into cerebellar, cortical and subcortical regions of interest (ROIs). Volumetric thickness maps for each participant were then generated using the DiReCT cortical thickness estimation method.^43^

Longitudinal cortical thickness measurement used the ANTs longitudinal pipeline.^44^ This method creates an unbiased within-participant template from all time points to allow consistent alignment across scans and reduce bias. Cortical thickness is then estimated using tissue segmentation in the native space.

### Plasma ptau_217_

All included individuals for T-N mismatch analyses had plasma ptau_217_ samples. All plasma samples were analysed on the Fujirebio Lumipulse G1200 analyser at either the University of Pennsylvania ADNI Biomarker Core (ADNI, Penn ADRC, ATM) or the University of Indiana Biomarker Assay Lab (ADNI). Within the ADNI cohort, 291 samples were analysed at the University of Pennsylvania and 156 at the University of Indiana. Tau positivity was defined as plasma ptau_217_ > 0.3175,^45^ with details described in the Supplementary material, ‘Plasma ptau_217_' section.

### Cognitive testing

Global clinical impairment was assessed using the CDRSB for both ADNI and Penn ADRC. Domain-level cognitive performance was derived from the ADSP Phenotype Harmonization Consortium’s composite scores dataset (ADSP_PHC_COGN_domain_wise_score) available through ADNI. This harmonized dataset provides standardized *z*-scores for cognitive domains, including memory, executive function and language.

### T-N mismatch modelling

To capture spatial patterns of T-N mismatch within the MTL, we used cortical thickness from the 100 super-points as a measure of N and plasma ptau_217_ as a measure of T. For each super-point, we performed robust linear regression^46^ between its corresponding thickness and ptau_217_ with Tukey’s bisquare function to downweight the influence of outliers. We then extracted the residuals as a measure of T-N mismatch. We also performed a separate regression of amygdala volume on ptau_217_, controlling for intracranial volume to obtain an additional residual reflecting mismatch in the amygdala. Residuals from both the super-points and amygdala were discretized into three categories (+1, 0, −1) based on whether they were >0.5 standard deviations (SD) above or below the regression line. Furthermore, the discretized residual for amygdala in each hemisphere was weighted seven times that of a single super-point residual, because the amygdala volume is approximately seven times the volume of a single super-point. The combined residual profiles were then clustered using Ward’s hierarchical clustering^47^ based on Euclidean distance to derive data-driven T-N mismatch phenotypes. The number of clusters was guided by visual inspection of the dendrogram (Supplementary Fig. 1) and informed by our prior work,^28-30^ which identified three MTL-involved groups at the whole-brain level, suggesting that a three-cluster solution might be optimal. This modelling approach was used to generate T-N groups in the ADNI cohort for the main analyses and replicated in an independent cohort from the Penn ADRC.

### Assigning clinical patients to ADNI-derived T-N phenotypes

As a proof of concept for clinical practice, we applied the T-N mismatch model trained on the ADNI cohort to an independent clinical dataset of patients undergoing anti-amyloid-β therapies (ATM cohort). For each patient in ATM, T-N residuals were computed at super-points and weighted amygdala regions using ADNI-derived regression models. These residuals were then binarized using a threshold of ±0.5 SD from the corresponding ADNI distribution. Each ATM patient was then matched to one of the three ADNI-derived T-N phenotypes by identifying the cluster centroid with the closest Euclidean distance in the binarized residual space. To facilitate broader application, we released a user-friendly software package for implementing the mismatch approach and individual T-N phenotypic classification (https://github.com/xueying-lyu/mismatch).

### Statistical modelling and analyses

#### Analyses overview

We first performed group comparisons between mismatch groups in the primary ADNI cohort to evaluate cross-sectional and longitudinal structural differences, in addition to differences in clinical and cognitive trajectories. We then replicated cross-sectional thickness comparisons and longitudinal cognitive impairment analyses in the Penn ADRC cohort. Finally, we performed vertex-wise cortical thickness comparisons in the MTL for the clinical ATM cohort. Details of these analyses are provided in later sections.

#### General statistical analyses

All general statistical analyses were performed using R (v.4.5). Between-group comparisons of continuous variables, including age, ptau_217_, imaging features and cognitive scores, were conducted using general linear models, with group as the independent variable and relevant covariates included for adjustment. The comparisons were two-sided. Ordinal variables, such as clinical diagnosis, were compared using the Kruskal–Wallis test.^48^

#### Cross-sectional thickness comparisons

##### MTL surface vertex-wise thickness comparisons

We performed vertex-wise comparisons of MTL thickness across T-N groups using a general linear model as implemented in the program meshglm (https://github.com/pyushkevich/cmrep). Prior to modelling, participant-level MTL thickness maps were smoothed using surface-based diffusion smoothing with a Gaussian kernel (σ ≈ 3 mm) applied on the mesh. For visualization, we fitted the CRASHS template to an ASHS segmentation of the MNI template^49^ and projected thickness map into this geometry, which more closely reflects typical MTL anatomy than the inflated CRASHS template. At each vertex of the template surface mesh, thickness was modelled as the dependent variable, group membership as the main factor of interest, and age, ptau_217_ and sex as covariates. We controlled for ptau_217_ to remove reduce effects of differences in tau severity between groups. For comparisons between T-N groups and A− cognitively unimpaired individuals, the model included age and sex as covariates. Group contrasts were computed at each vertex, and *t*-statistics were derived. To correct for multiple comparisons, we applied permutation testing with 10 000 iterations using threshold-free cluster enhancement,^50^ with parameters extent weight ()= 0.5, height weight (*H*) = 2 and step size (Δ*h*) = 0.1, yielding family-wise error rate^51^ (FWER)-corrected *P*-values at each vertex.

##### Whole-brain voxel-wise thickness comparisons

Additionally, voxel-wise comparisons of whole-brain grey matter thickness across groups were performed with FMRIB Software Library (FSL)’s randomize tool using threshold-free cluster enhancement for 10 000 permutations, controlling for age, sex and ptau_217_. For comparisons between T-N groups and A− cognitively unimpaired individuals, age and sex were included as covariates. Likewise, the *P*-values were corrected by FWER.

#### Longitudinal thickness analyses

##### MTL vertex-wise atrophy

We performed vertex-wise linear mixed-effects modelling^52^ using the lme4 package in R, with longitudinal MTL thickness as the dependent variable, Group × Time interaction as the fixed effect, and participant as the random effect, controlling for age, ptau_217_ and sex. We restricted the time from baseline to a ±5 year window. Among 447 ADNI participants, 370 had longitudinal MRI scans, with an average of 4.08 ± 1.80 scans per participant over an average follow-up span of 3.49 ± 1.96 years. The 123 A− cognitively unimpaired controls from ADNI had 3.3 ± 1.34 time points over 4.07 ± 1.85 years of follow-up. Differences in thickness changes across groups were determined by testing the significance of the Time × Group interaction term. Comparisons with cognitively unimpaired controls were adjusted for age and sex. Group-wise *t*-statistics and false discovery rate (FDR)-corrected *P*-values were computed.

##### Whole brain voxel-wise atrophy

To assess whole-brain longitudinal atrophy differences between groups, we applied voxel-wise linear mixed-effects modelling using lmerNIFTI.^53^ We included time points from baseline between −5 and 5 years. Owing to the computational limitation, for participants with more than two scans, we required that each scan be ≥1 year apart. Of the 370 ADNI T-N patients with longitudinal MRI scans, seven failed whole-brain longitudinal processing, resulting in 363 patients with a mean of 3.03 ± 1.06 time points over an average follow-up period of 3.36 ± 2.01 years. The 123 A− cognitively unimpaired controls from ADNI had 2.95 ± 0.96 time points over 4.02 ± 1.83 years of follow-up. Longitudinal cortical thickness was modelled as the dependent variable, with Group × Time interaction as the fixed effect and with participant as the random intercept, controlling for age, ptau_217_, and sex. For the comparisons of groups with cognitively unimpaired controls, we controlled only for age and sex. Differences in thickness changes across groups were determined by testing the significance of the Time × Group interaction term. Resulting *P*-values were corrected for multiple comparisons using FDR.

##### Longitudinal atrophy preceding tau positivity

For analyses focused on the period preceding estimated Tau positivity (T+; see Supplementary material, ‘Estimated tau onset age using SILA' section), we restricted the data to the interval from −15 to 0 years relative to estimated T+ age. Among the included 447 A+ patients from ADNI, there were 172 A+ individuals with longitudinal MRI scans before estimated tau onset, with a mean of 2.84 ± 1.04 time points over 3.05 ± 2.00 years. We again conducted both longitudinal MTL and whole-brain voxel-wise analyses based on linear mixed-effects models following the same procedures described above. Similarly, the models included the interaction between time-from-T+ and group as the fixed effect, but with age at estimated T+ and sex as covariates.

#### Longitudinal cognitive impairment analyses

##### Modelling longitudinal cognitive trajectories relative to tau positivity

Cognitive trajectories were analysed using linear mixed-effects models,^52^ with cognitive scores as the outcome. We included cognitive time points within a ±5 year window relative to the estimated T+ time (Supplementary material, ‘Estimated tau onset age using SILA' section). The models included fixed effects for Time × Group interaction (using b-spine with two degrees of freedom), in addition to covariates for age at estimated T+, sex and education. A random intercept was included to account for within-participant variability. Differences in cognitive decline across groups were determined by testing the significance of the Time × Group interaction term. All statistical tests were two-sided. The *P*-values were Bonferroni-corrected for multiple comparisons. We also repeated the same modelling procedure with time anchored to baseline instead of estimated T+, including ptau_217_, age at baseline, sex and education as covariates.

## Results

### T-N mismatch groups

In order for our T-N mismatch measurements to be meaningful, a significant association between T and N must be observed across most of the MTL. To assess this, we first examined vertex-wise associations between plasma ptau_217_ and MTL regional thickness among 447 A+ individuals from ADNI (Fig. 1A). The level of ptau_217_ was significantly associated with reduced cortical thickness throughout the entire MTL area, with the strongest effects observed in hippocampal and anterior extrahippocampal areas, after adjusting for age and sex.

**Figure 1:**
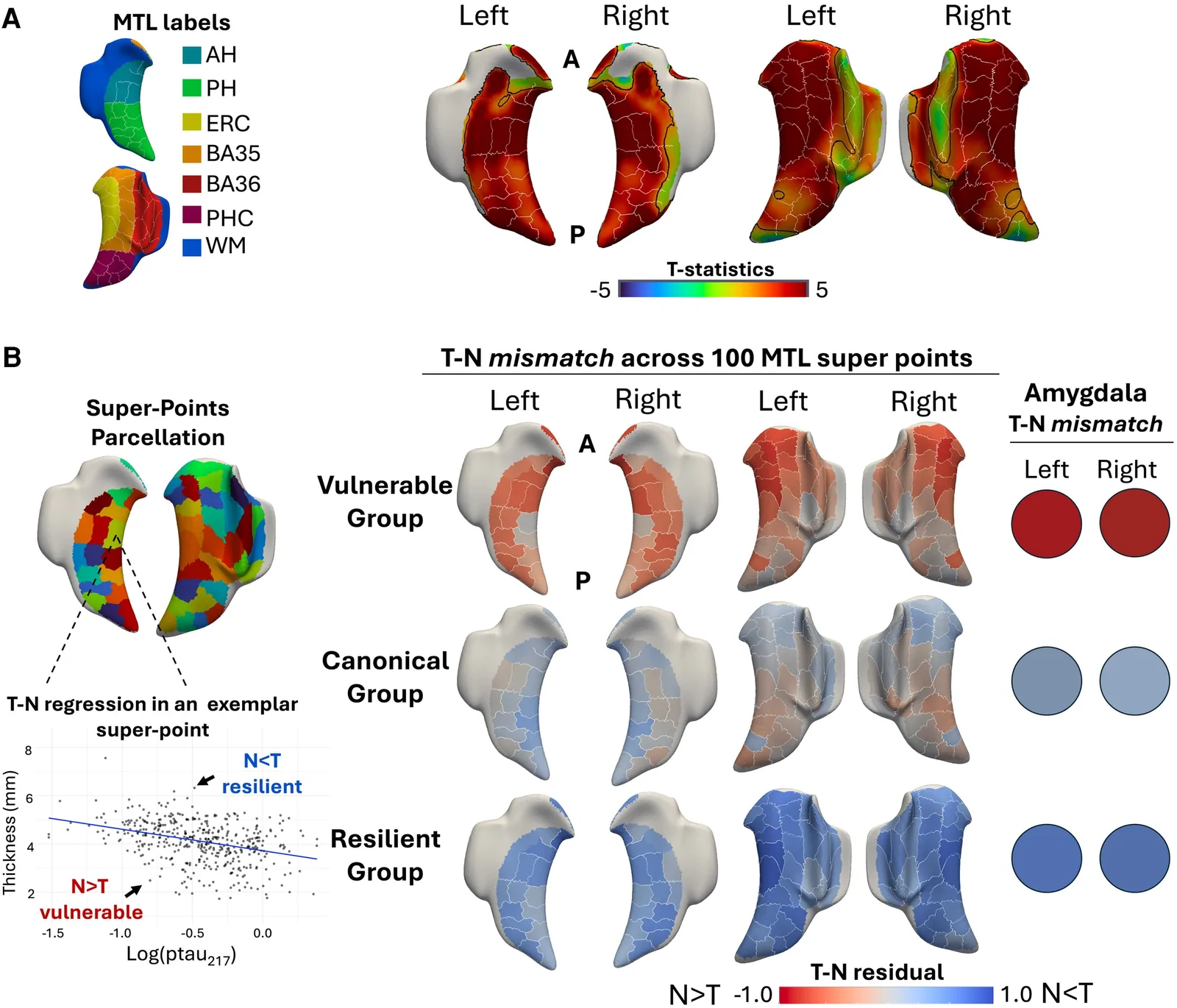
**Medial temporal lobe T-N mismatch.** (**A**) Vertex-wise association between MTL thickness and ptau_217_ for A+ individuals from the Alzheimer’s Disease Neuroimaging Initiative after adjusting for age and sex. The white edges represent the outline of parcellated super-points. The black edges indicate the area with significant association (*P*_FWER_ < 0.05). (**B**) Visualization of T-N residual patterns, calculated as standardized residuals (residuals divided by their standard deviation), across 100 super-points within the MTL and amygdala for A+ individuals. AH = anterior hippocampus; BA35 = Brodmann area 35; BA36 = Brodmann area 36; ERC = entorhinal cortex; MTL = medial temporal lobe; N = neurodegeneration; PH = posterior hippocampus; PHC = parahippocampal cortex; T = tau; WM = white matter.

We next performed T-N mismatch clustering based on deviation from the relationship of ptau_217_ to cortical thickness from 100 MTL super-points and amygdala volume, identifying three T-N subgroups. The ‘canonical’ group (32.4%) displayed minimal T-N residuals across the MTL and amygdala, reflecting a relatively close alignment between tau burden and neurodegeneration (T ∼ N). The ‘vulnerable’ group (36.9%) exhibited negative T-N residuals, with greater thinning than expected based on ptau_217_ levels, particularly in the amygdala and anterior MTL regions (N > T). In contrast, the ‘resilient’ group (30.6%) displayed positive T-N residuals, suggesting relative preservation of structure despite elevated tau, most prominently in the amygdala and diffuse extrahippocampal areas (T > N). These groups were relatively balanced in size and exhibited distinct patterns of T-N mismatch (Fig. 1B). A significant difference in ptau_217_ was observed, with the vulnerable group showing a higher ptau_217_ level than the canonical group (*P* = 0.039; Table 1). All subsequent analyses were adjusted for ptau_217_ to account for this difference.

**Table 1:** Comparisons of demographic and other characteristics between T-N groups for amyloid-β-positive individuals (*n* = 447) from the Alzheimer’s Disease Neuroimaging Initiative

	Basic characteristics Covariates for ptau_217_ and centiloid: age and sex					Imaging features Covariates: ptau_217_, age, sex and ICV (ICV not included for ERC:PHC)				Cognition Covariates: ptau_217_, age, sex and years of education			
Description	Age, years	Sex, F/M	ptau_217_	Centiloid	Diagnosis CU/MCI/Dem	AH volume, mm^3^	PH volume, mm^3^	ERC:PHC ratio	Amyg volume, mm^3^	CDRSB	MEM	LAN	EXF
Group 1 Vulnerable (N > T) (*n* = 165)	77.6 (7.6)	85/80	0.509 (0.39)*	81.6 (39)	25/56/84	1415 (274)***	1359 (284)***	0.923 (0.089)***	964 (167)***	4.09 (3.6)***	−0.46 (0.9)***	−0.050 (0.7)***	−0.15 (0.8)***
Group 2 Canonical (N ∼ T) (*n* = 145)	76.2 (7.3)	73/72	0.409 (0.36)	72.9 (37)	72/47/26	1689 (249)	1554 (168)	1.04 (0.081)	1183 (136)	1.61 (2.8)	0.37 (0.9)	0.47 (0.8)	0.35 (0.8)
Group 3 Resilient (N < T) (*n* = 137)	74.3 (7.9)	62/75	0.504 (0.39)	76.4 (39)	66/56/15	1814 (249)***	1665 (190)***	1.01 (0.079)	1236 (137)**	1.05 (1.5)**	0.51 (0.7)**	0.55 (0.6)*	0.33 (0.7)
Group difference	*P* < 0.01	*P* = 0.53	*P* = 0.039	*P* = 0.44	*P* < 0.001	*P* < 0.001	*P* < 0.001	*P* < 0.001	*P* < 0.001	*P* < 0.001	*P* < 0.001	*P* < 0.001	*P*< 0.001

Overall group effects (*P*-values indicated in the bottom row) were tested using the Kruskal–Wallis test for categorical variables and linear regression for continuous variables. Covariates for comparisons are listed in the table. Only significant pairwise comparisons between vulnerable and resilient groups with the canonical group are marked in the table. All comparisons were two-sided. The *P*-values were Bonferroni-corrected for multiple comparisons (*P < 0.05, **P < 0.01 and ***P < 0.001). AH = anterior hippocampus; Amyg = amygdala; CDRSB = Clinical Dementia Rating Sum of Boxes; Dem = dementia; ERC = entorhinal cortex; EXF = executive function; F = female; ICV = intracranial volume; LAN = language; M = male; MEM = memory; PH = posterior hippocampus; PHC = parahippocampal cortex.

The demographics of these T-N groups are shown in Table 1. The groups differed in age (*P* < 0.05), with the vulnerable group tending to be older than the canonical and resilient groups. There were no differences in sex distribution across groups (*P* = 0.53).

### Cross-sectional and longitudinal atrophy differences between T-N groups

We next compared structural differences among ADNI T-N groups. Although we did observe some increased ptau_217_ level in the vulnerable and resilient groups relative to the canonical (*P* = 0.039), we observed a significant difference in regional structure (Table 1) after controlling for ptau_217_, age, sex and intracranial volume. The vulnerable group had significantly reduced amygdala volume compared with the canonical group (*P* < 0.001), and the resilient group had a larger volume (*P* < 0.01). The vulnerable group displayed a significantly lower volume in both anterior and posterior hippocampal regions (*P* < 0.001), whereas the resilient group demonstrated greater volume (*P* < 0.001). Additionally, the T-N groups differed in the entorhinal cortex-to-parahippocampal cortex (ERC:PHC) ratio, which is an imaging feature suggestive of comorbid LATE in AD (Supplementary material, ‘Imaging signature of LATE' section), with the vulnerable group showing a significantly lower ratio than the others (*P* < 0.001).

To characterize structural differences further, we performed vertex-wise cortical thickness comparison across the MTL (Fig. 2A). In line with T-N mismatch patterns and regional volume results, the vulnerable group exhibited significantly lower thickness than the canonical group, prominently in the anterior extrahippocampal and hippocampal regions within the MTL. In contrast, the resilient group displayed greater thickness in widespread MTL regions. Extending to the whole-brain comparisons (Fig. 2B), these patterns were further supported, with the vulnerable group showing anterior temporal-limbic and orbito-frontal thinning relative to the canonical group, whereas the resilient group exhibited preservation of cortical thickness in temporal lobe and posterior cortices.

**Figure 2:**
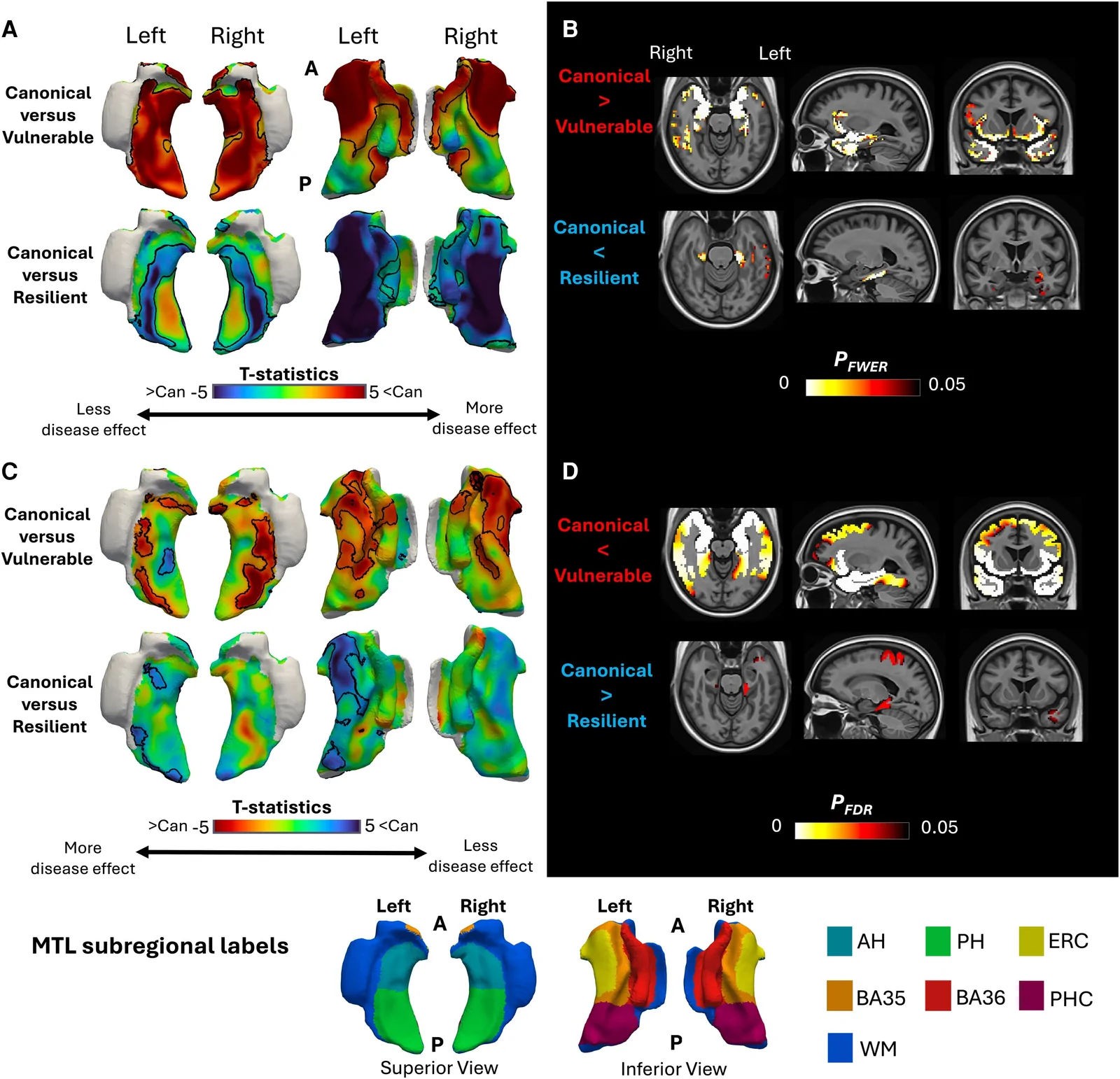
**Structural differences between T-N groups.** (**A** and **B**) Cross-sectional comparisons after controlling by ptau_217_, age and sex. (**A**) T-statistics for vertex-wise thickness comparisons: canonical versus vulnerable group in the *top row* and canonical versus resilient group in the *bottom row*. Positive *t*-values indicate less thickness for the vulnerable/resilient group compared with the canonical group. The black edges highlight the area with significant thickness comparisons (*P*_FWER_ < 0.05). (**B**) Voxel-wise thickness comparisons in whole brain between the vulnerable and canonical groups (*top*) and between resilient and canonical groups (*bottom*). The coloured areas represent significant differences for the vulnerable and resilient groups with the canonical group (*P*_FWER_ < 0.05). (**C** and **D**) Longitudinal comparisons after controlling by ptau_217_, age and sex. (**C**) The *t*-statistics for vertex-wise longitudinal atrophy comparisons using linear mixed-effects models are shown: canonical versus vulnerable (*top*) and canonical versus resilient (*bottom*). Negative *t*-values indicate greater atrophy in the comparison group relative to the canonical group. The black edges represent significant differences with the canonical group for each T-N mismatch group (*P*_FDR_ < 0.05). (**D**) Voxel-wise longitudinal cortical thickness changes across the whole brain using linear mixed-effects models: canonical versus vulnerable (*top*) and canonical versus resilient (*bottom*). Coloured area indicates voxels with significant differences in atrophy rates compared to the canonical group (*P*_FDR_ < 0.05). A = anterior; AH = anterior hippocampus; BA35 = Brodmann area 35; BA36 = Brodmann area 36; Can = canonical; ERC = entorhinal cortex; FDR = false discovery rate; FWER = family-wise error rate; MTL = medial temporal lobe; N = neurodegeneration; P = posterior; PH = posterior hippocampus; PHC = parahippocampal cortex; T = tau; WM = white matter.

Longitudinal results were consistent with cross-sectional patterns (Fig. 2C and D). In comparison to the canonical group, the vulnerable group displayed greater thinning over time, particularly in the anterior extrahippocampal and anterior temporal regions. In contrast, the resilient group exhibited slower cortical thinning relative to the canonical group.

In addition to the comparisons between T-N groups, we also compared the structural differences of the ADNI T-N groups with A− cognitively unimpaired group (Supplementary Fig. 2). The vulnerable group exhibited the most extensive atrophy, both within the MTL and across the whole brain. The canonical group showed a more uniform pattern of atrophy within the MTL, along with AD-like posterior cortical thinning. The resilient group demonstrated only focal significant changes both cross-sectionally and longitudinally, with a mild AD-like atrophy pattern.

### The vulnerable group exhibited early atrophy prior to predicted onset time of tau positivity

We next examined longitudinal structural changes up to 15 years preceding estimated tau onset across T-N groups in ADNI (Fig. 3). We observed that the vulnerable group displayed significantly greater thinning in anterior extrahippocampal regions of the MTL and broader limbic areas across the brain, compared with the canonical group. The resilient group exhibited no significant differences from the canonical group (Fig. 3).

**Figure 3:**
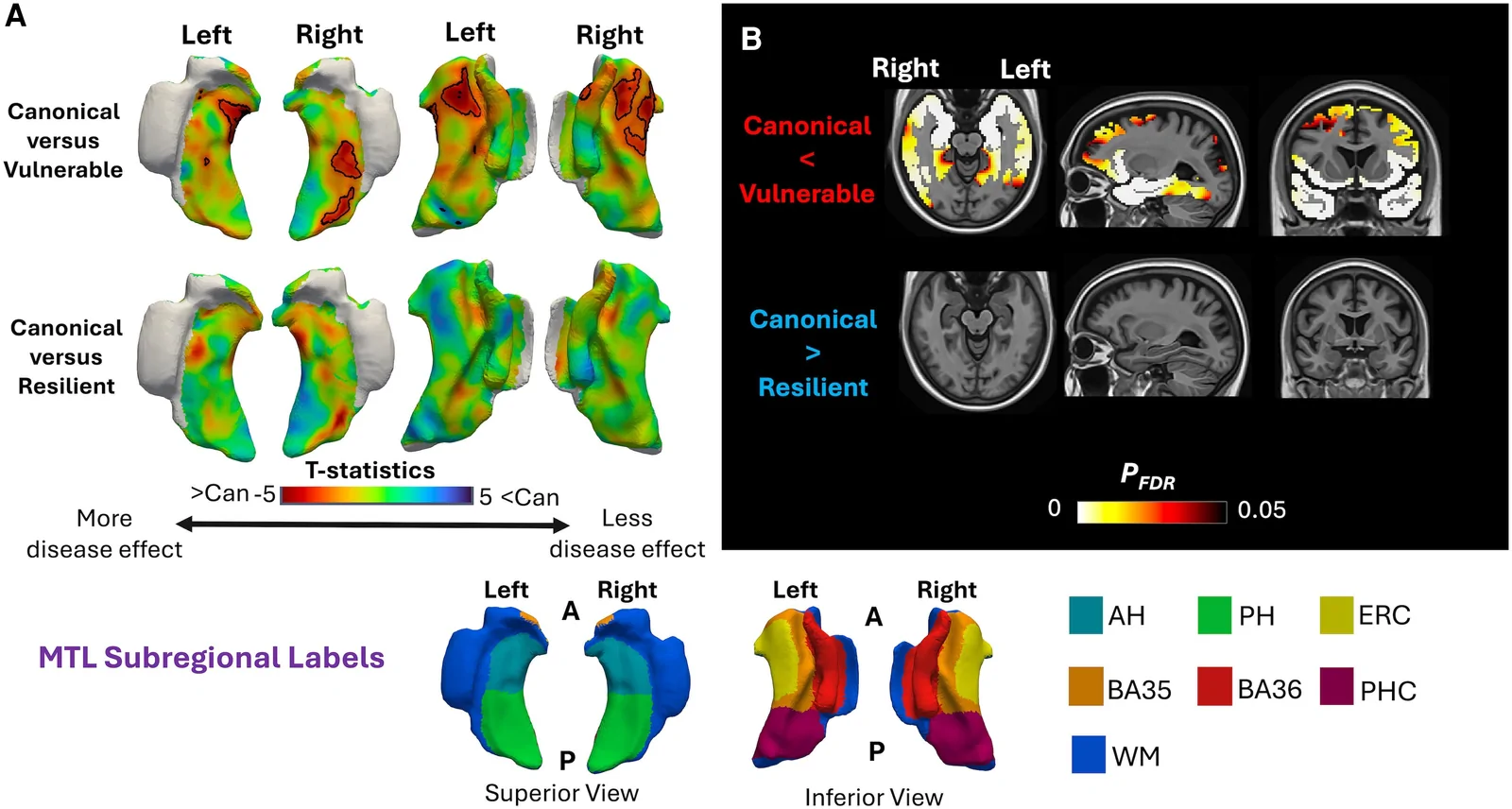
**Early structural changes before estimated tau onset.** (**A**) Vertex-wise *t*-maps comparing pre-tau onset atrophy between canonical and mismatch groups (vulnerable, *top*; resilient, *bottom*), adjusted for estimated tau onset age and sex. Negative *t*-values indicate greater atrophy relative to the canonical group. The black edges highlight the area with significant thickness comparisons (*P*_FDR_ < 0.05). (**B**) Voxel-wise comparisons of whole-brain cortical thinning before tau positivity between groups (*P*_FDR_ < 0.05), adjusted for estimated tau onset age and sex. AH = anterior hippocampus; BA35 = Brodmann area 35; BA36 = Brodmann area 36; Can = canonical; ERC = entorhinal cortex; FDR = false discovery rate; MTL = medial temporal lobe; N = neurodegeneration; PH = posterior hippocampus; PHC = parahippocampal cortex; T = tau; WM = white matter.

### T-N groups displayed distinct cognitive profiles

At baseline, the vulnerable group displayed a significantly higher CDRSB score (*P* < 0.001) compared with the canonical group, whereas the resilient group had a lower (*P* < 0.01) mean CDRSB measure (Table 1). The groups also differed in domain-specific cognitive functions. The vulnerable group displayed significantly worse performance in memory, language and executive function relative to the canonical group (*P* < 0.001). In contrast, the resilient group demonstrated better performance in memory (*P* < 0.01) and language (*P* < 0.05).

We further evaluated longitudinal cognitive trajectories relative to estimated T+ onset age across the T-N groups. Change in CDRSB, memory, language and executive function over time were modelled after adjusting for age at T+ onset, sex and years of education (Fig. 4). The vulnerable group exhibited significantly faster increase in CDRSB scores compared with the canonical group (*P* < 0.001). At the domain level, the vulnerable group demonstrated greater decline in memory (*P* < 0.001), language (*P* < 0.01) and executive function (*P* < 0.001) relative to the canonical group, although with the greatest differences in memory in absolute terms. Notably, the vulnerable group showed signs of cognitive impairment, especially in memory, prior to estimated T+ (Fig. 4). We did not observe a significant difference between the resilient group and the canonical group in cognitive trajectories. We also modelled longitudinal cognitive trajectories anchored to baseline rather than T+ time and observed similar patterns (Supplementary Fig. 3).

**Figure 4:**
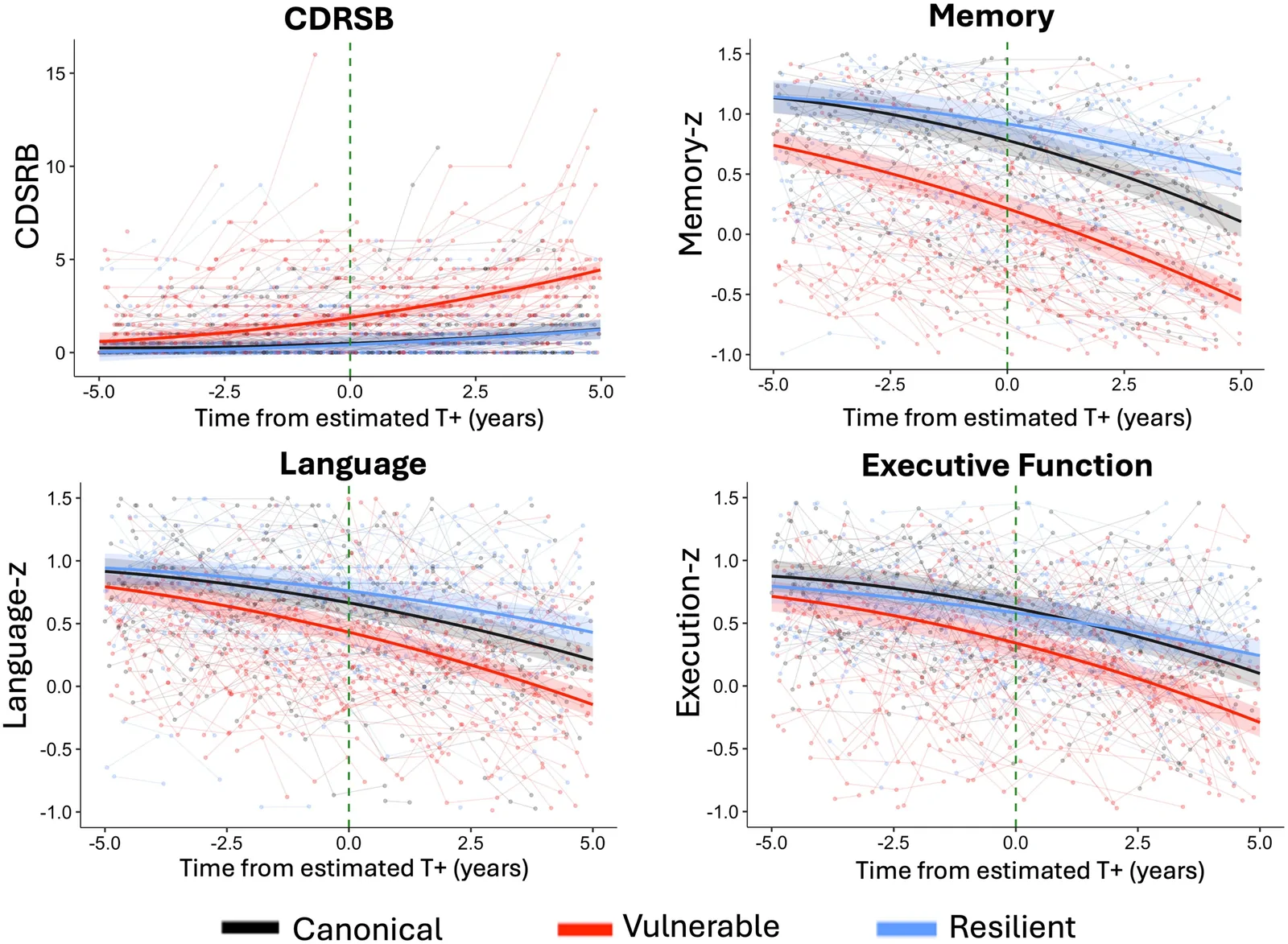
**Cognitive trajectories anchored to estimated tau onset across T-N groups.** Longitudinal cognitive trajectories aligned to estimated T+ across CDRSB, memory, language and executive function *z*-scores between T-N groups. Shaded ribbons indicate 95% confidence intervals for model-predicted trajectories, while semi-transparent spaghetti lines represent individual individual-level cognitive trajectories over time. The Group × Time effects were adjusted by estimated tau onset age, sex and years of education. CDRSB = Clinical Dementia Rating Sum of Boxes; T+ = tau onset.

### T-N groups were replicated in an independent cohort

To evaluate the robustness of our approach, we replicated the analysis in an independent cohort of A+ individuals, including cognitively unimpaired, MCI and dementia-level impairment (Supplementary Table 1) from the Penn ADRC. Once again, we identified three distinct T-N groups (Fig. 5A): vulnerable (N > T, 12%), canonical (N ∼ T, 53.7%) and resilient (N < T, 34.3%). The vulnerable group, similar to the ADNI cohort, displayed disproportionate neurodegeneration relative to tau burden in the amygdala, hippocampus and anterior extrahippocampal areas (Fig. 5A). In contrast, the resilient group displayed neurodegeneration less than expected for T in a broad pattern across the MTL, and the canonical group had low T-N residuals across MTL super-points and the amygdala.

**Figure 5:**
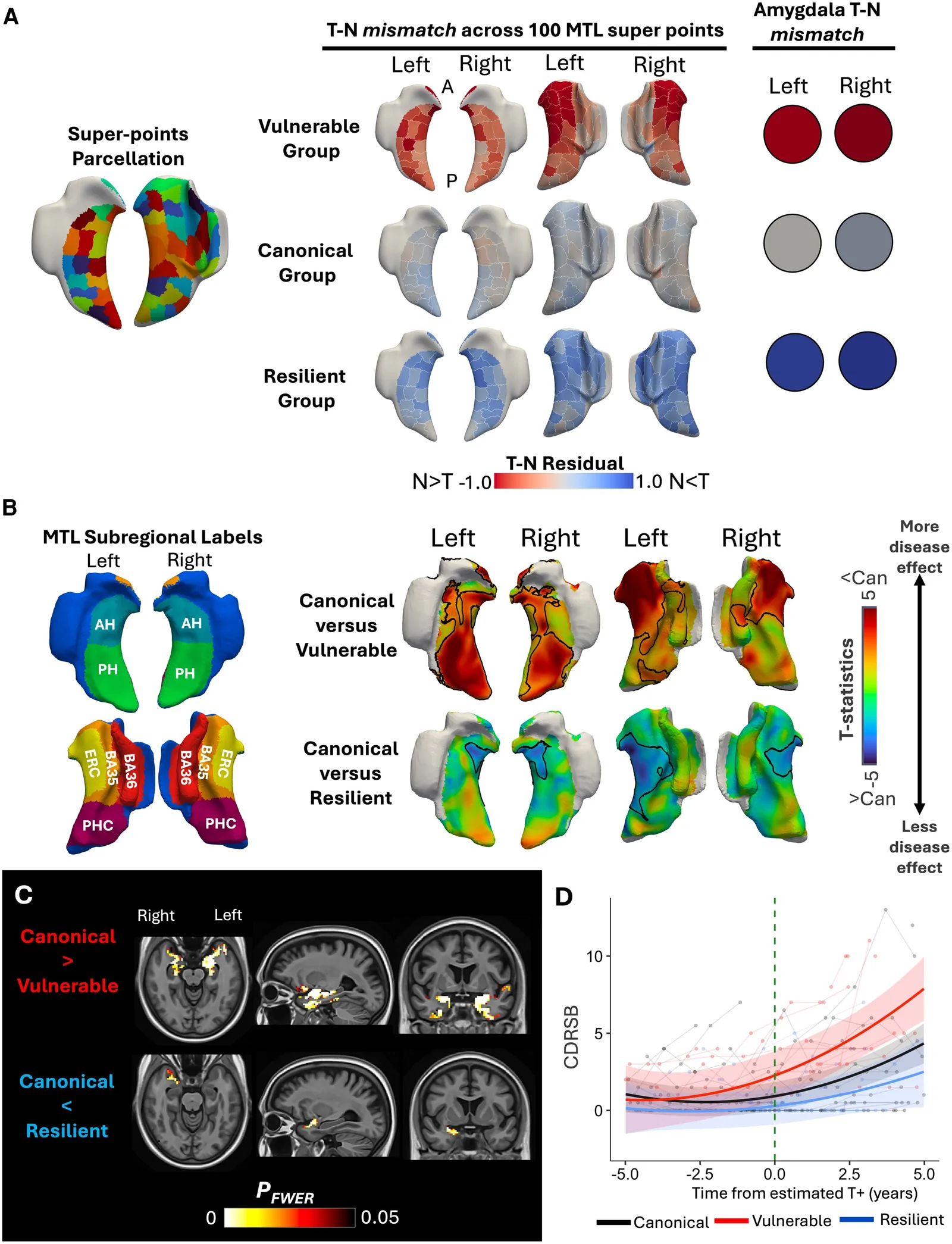
**Replication of MTL T-N mismatch from Penn ADRC dataset.** (**A**) Visualization of T-N residual patterns across 50 bilateral super-points within the MTL and amygdala for 108 A+ individuals from the Penn ADRC. The white edges represent the outline of parcellated super-points. (**B**) The *t*-statistics are shown for vertex-wise thickness comparisons among T-N groups from the Penn ADRC cohort: canonical versus vulnerable group in the *top row* and canonical versus resilient group in the *bottom row*. Comparisons were adjusted for ptau_217_, age and sex. Negative *t*-values indicate less thickness for the vulnerable/resilient group compared with the canonical group. The black edges highlight the area with significant thickness comparisons (*P*_FWER_ < 0.05). (**C**) Voxel-wise whole-brain thickness comparisons between the vulnerable group and the canonical group (*top*) and between the resilient group and the canonical group (*bottom*). Comparisons were adjusted by ptau_217_, age and sex. The coloured areas represent significant differences with the canonical group for each T-N mismatch group (*P*_FWER_ < 0.05). (**D**) Longitudinal cognitive trajectories relative to estimated T+ for CDRSB between T-N groups. Shaded ribbons indicate 95% confidence intervals for model-predicted trajectories, while semi-transparent spaghetti lines represent individual participant-level cognitive trajectories over time. The Group × Time effects were adjusted by estimated tau onset age, sex and years of education. ADRC = Alzheimer’s Disease Research Center; AH = anterior hippocampus; BA35 = Brodmann area 35; BA36 = Brodmann area 36; Can = canonical; CDRSB = Clinical Dementia Rating Sum of Boxes; ERC = entorhinal cortex; FWER = family-wise error rate; MTL = medial temporal lobe; N = neurodegeneration; PH = posterior hippocampus; PHC = parahippocampal cortex; T = tau; T+ = tau onset; WB = whole brain; WM = white matter.

Consistent with findings from the ADNI cohort, the vulnerable group in the Penn ADRC cohort exhibited a lower ERC:PHC ratio (*P* < 0.001), reduced amygdala volume (*P* < 0.001) and greater hippocampal atrophy (Supplementary Table 2). Within the MTL, this group displayed significantly reduced cortical thickness in both the hippocampus and specifically anterior extrahippocampal regions compared with the canonical group (Fig. 5B). Whole-brain analysis further revealed more extensive thinning in anterior temporal-limbic regions (Fig. 5C). The resilient group showed more thickness preservation in the extrahippocampal region. Also similar to the ADNI cohort, the vulnerable group tended to exhibit higher baseline CDRSB scores and faster longitudinal cognitive decline (Fig. 5D), although this did not reach statistical significance.

### Applicability of T-N mismatch to clinical practice

Finally, we evaluated the applicability of this approach in a real-world clinical cohort (ATM) of 50 patients with AD who underwent anti-amyloid therapy. A schematic diagram illustrating the workflow for an exemplar patient is shown in Fig. 6A. Using the T-N models derived from ADNI, we predicted individual T-N mismatch profiles across MTL super-points and amygdala in the ATM cohort. Each patient was then assigned to the most similar T-N phenotype by computing the Euclidean distance between their residual pattern and the centroid of each ADNI-derived cluster and selecting the phenotype corresponding to the closest distance. After assignment (Fig. 6B), 44% of patients were classified as canonical (*n* = 22), 36% as vulnerable (*n* = 18) and 20% as resilient (*n* = 10). This distribution was similar to the T-N group proportions observed in the ADNI cohort. We then examined group-level characteristics following this assignment (Supplementary Table 3). Despite the vulnerable group having lower ptau_217_ levels than the canonical group (*P* < 0.01), it demonstrated lower volumes in the amygdala (*P* < 0.001) and hippocampus (*P* < 0.001), especially in the anterior hippocampus, compared with the canonical group, after adjusting for ptau_217_, age, sex and intracranial volume. In contrast, the resilient group showed higher hippocampal volume (*P* < 0.05). Differences were also observed in the ERC:PHC ratio (*P* < 0.01), with the vulnerable group exhibiting a lower ratio (*P* < 0.05). Furthermore, vertex-wise comparisons revealed reduced thickness in the anterior extrahippocampal regions in the vulnerable group relative to the canonical group (Fig. 6C), whereas the resilient group showed greater thickness. These findings closely align with those observed in the ADNI-defined T-N groups and the Penn ADRC replication cohort.

**Figure 6:**
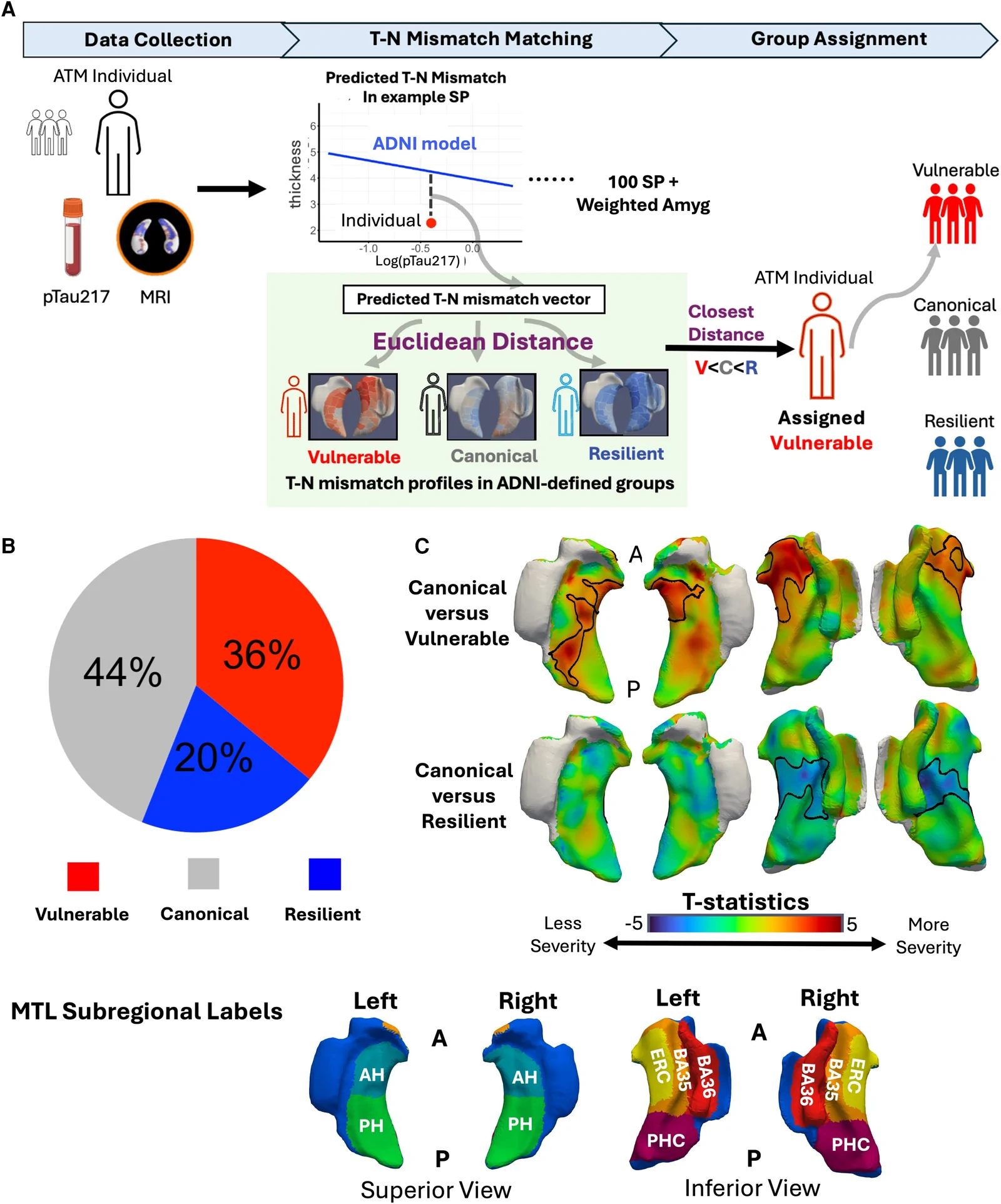
**Application of MTL T-N mismatch model to 50 patients treated with anti-amyloid therapeutics.** (**A**) Schematic diagram illustrating group assignment for a representative individual based on T-N mismatch. (**B**) Pie chart showing the distribution of assigned T-N groups among the 50 individuals. (**C**) Vertex-wise thickness comparisons between T-N groups in the ATM cohort: canonical versus vulnerable (*top*) and canonical versus resilient (*bottom*), adjusted for ptau_217_, age and sex. Negative *t*-values indicate lower thickness in vulnerable/resilient groups. Black outlines denote regions with significant differences (*P*_FWER_ < 0.05). AH = anterior hippocampus; Amyg = amygdala; BA35 = Brodmann area 35; BA36 = Brodmann area 36; C = canonical; ERC = entorhinal cortex; FWER = family-wise error rate; MTL = medial temporal lobe; N = neurodegeneration; PH = posterior hippocampus; PHC = parahippocampal cortex; R = resilient; SP = super-points; T = tau; V = vulnerable; WM = white matter.

## Discussion

AD is heterogeneous, with neurodegeneration often influenced by co-pathologies and resilience factors. Given the present limitations of *in vivo* biomarkers for detecting co-pathologies, indirect approaches to infer their presence and severity are valuable for prognosis and potentially for determining appropriate patients for targeted therapies. We demonstrated that mismatch between tau and neurodegeneration across the whole brain identified individuals who were likely to harbour co-pathologies (e.g. TDP-43, vascular burden) that might contribute to greater vulnerability and a faster rate of cognitive decline. Additionally, this approach also classified some individuals as resilient with a more indolent rate of decline, as shown previously.^28-30^

The present analysis focused specifically on the MTL, a region associated not only with the early neurofibrillary tangle pathology of AD, but also with one of its most common co-pathologies, LATE-NC, which is present in ≤50% of patients with AD.^17^ Previous work has demonstrated that when AD co-occurs with LATE-NC, patients have a faster rate of cognitive decline and hippocampal atrophy.^10,54-56^ Thus, identification of such patients has particular clinical significance in practice and clinical trials. LATE-NC does not affect the MTL uniformly and has a spatial pattern that does not completely overlap with typical AD. Specifically, LATE-NC displays an anterior-to-posterior gradient of atrophy, including early involvement of the amygdala (stage 1), the anterior hippocampus and the entorhinal cortex (stage 2).^18,20,23^ In contrast, typical AD tends to involve the posterior MTL to a greater extent than LATE, particularly the parahippocampal cortex,^18^ although both AD and LATE exhibit a degree of predilection for the anterior MTL.^10,35^ To capture these spatial differences, we parcellated the MTL into 100 super-points and incorporated the amygdala for volume-based weighted T-N mismatch clustering.

To increase the potential clinical accessibility of the approach described, we used plasma ptau_217_, as a global measure of tau pathology, as opposed to tau PET. The latter provides important spatial information, but is currently not generally available in clinical practice, limited by variable insurance coverage, cost and access to PET scanners. Plasma measures offer a much less expensive alternative. Previous studies^45,57,58^ demonstrated that although plasma ptau_217_ is highly sensitive to early amyloid-β status, it also has a strong correlation with both temporal and global measures of tau burden measured by tau PET. Indeed, in the context of individuals who are A+, the relationship to tau PET is generally significantly stronger than amyloid PET.^58^ Regardless, it is reasonable to conceptualize ptau_217_ as an overall measure of AD biological severity, where neurodegenerative mismatch with this measure reflects additional non-AD factors.

Using this approach, we identified three data-driven T-N groups, which were labelled as vulnerable (N > T), canonical (N ∼ T) or resilient (N < T), among patients on the AD continuum. The vulnerable group displayed a number of features suggestive of the presence of LATE-NC, including more severe atrophy in anterior MTL regions, as evidenced by a lower ERC:PHC ratio, a structural signature previously found to be associated with AD with concomitant TDP-43 in post-mortem studies.^18^ Additionally, this group displayed a pattern of atrophy outside the MTL in anterior temporal-limbic areas and fronto-insular and orbitofrontal cortices that reflect regions of TDP-43 progression in LATE-NC.^56,59^ Longitudinal patterns of atrophy largely overlapped with the cross-sectional findings.

In addition to the imaging features suggesting concomitant LATE-NC in the vulnerable group, its cognitive profile was also consistent with studies of mixed AD and LATE-NC. In particular, memory impairment is the hallmark clinical feature of LATE-NC, and semantic memory/language may also be involved.^10,17,60^ Consistent with this pattern and a faster rate of decline than typical AD, the vulnerable group displayed most prominently a faster rate of decline in memory followed by language and executive measures.

Interestingly, the vulnerable group showed LATE-like atrophy patterns even before reaching estimated T+, in addition to evidence of progressive memory impairment prior to T+. In light of the strong relationship between development of tau pathology and cognitive decline,^61-63^ this finding suggests an independent, non-AD process also contributing to decline in this group, providing further support for LATE-NC as a contributor in these cases. It also suggests that some cases that are A+ might have LATE-NC as an earlier driver of symptoms prior to the evolution of AD pathology.^64^

In contrast, the resilient group, defined by less neurodegeneration than expected given their tau burden, showed greater preservation of thickness and slower rates of atrophy in the MTL and whole brain. In our prior work^28-30^ examining whole-brain T-N mismatch younger age was found to be a potential protective factor associated with the resilient phenotype. However, in the present study, where resilience was defined based on MTL-specific T-N mismatch, only a modest age difference was observed, suggesting that distinct or additional protective mechanisms might be involved. One possibility is that these individuals might be particularly free from co-pathologies, whereas the canonical group might harbour an intermediate burden. Future research is needed to gain a better understanding of the underlying factors contributing to resilience in this group.

We reproduced the T-N groups and observed consistent structural and cognitive patterns across groups in an independent research dataset. The vulnerable group again demonstrated features suggestive of LATE-NC involvement, including reduced ERC:PHC ratio, lower amygdala volume and anterior MTL atrophy, along with accelerated cognitive decline. The resilient group remained cognitively preserved despite tau burden. These replicated findings support the potential utility of T-N mismatch modelling as a reliable and generalizable *in vivo* approach for identifying susceptibilities and resilience within the AD continuum.

Importantly, we extended this approach to a real-world clinical cohort of patients with AD undergoing anti-amyloid therapy, representing the first application of this T-N mismatch framework beyond research cohorts. The observation of similar T-N group patterns in this clinical dataset supports the generalizability and translational potential of this approach. Among the 50 treated AD patients, only 44% were classified as canonical, while the remaining 56% exhibited divergent T-N mismatch patterns, including 36% identified as vulnerable and 20% as resilient. Consistent with the findings from ADNI, vulnerable individuals exhibited a lower ERC:PHC ratio compared with the canonical and resilient groups, with the value similar to what was observed in the ADNI vulnerable group. These findings highlight the potential of the approach for disentangling the heterogeneity in treatment populations, and it will be important to determine how these groups respond to such treatments in this context. For example, if the canonical or resilient group harbour less co-pathology, they might show the most robust effects of an amyloid specific therapy.

Although categorical classification has clinical value potentially mapping on to specific processes, such as LATE, it might also be the case that a more continuous measure of vulnerability or resilience might capture severity, which might also be important to quantify. To address this, we performed an additional analysis in which we derived a global MTL mismatch metric by summing discretized T-N residual values of super-points classified as vulnerable (+1), canonical (0) or resilient (−1), with higher values indicating greater vulnerability and lower values indicating greater resilience. The mismatch metric value showed significant associations with lower ERC:PHC thickness ratio, in addition to greater cognitive decline across multiple domain functions (memory > language > executive functioning) after adjusting for age, sex and ptau_217_ (Supplementary Fig. 5). These findings support a continuum of T-N mismatch severity that might reflect differences in the burden or stage of co-pathology. A limitation of this global metric is that it collapses spatial information, potentially conflating distinct anatomical patterns of vulnerability or resilience. More spatially specific residual measures, for example focused on anterior MTL regions for LATE, might provide additional insight in future studies.

Our work has limitations. The choice of parameters, such as the number of super-points and the number of T-N groups, was made empirically. Although super-points were generated strictly within anatomically defined regions of interest, with no super-point crossing regional boundaries, the variation in super-point parcellation boundaries might also introduce noise in later group classification. To assess robustness to boundary variability, we performed a spatially permuted cross-validation study in which the super-point parcellation was regenerated across 10 folds while preserving the number and approximate size of super-points (Supplementary Fig. 4A). T-N mismatch clustering was repeated for each fold and showed largely consistent group assignments (Supplementary Fig. 4B). A consensus T-N grouping derived by majority voting across folds agreed with the current classification in 83.8% of participants. Importantly, stability was greatest for vulnerable and resilient individuals (Supplementary Fig. 4C). Therefore, although boundary variability might have some mild influence on categorization, these results suggest that the identified T-N groups are generally stable to perturbations in super-point boundaries. It is worth noting that our primary motivation of using the super-point parcellation of regions of interest here was to reduce potential bias introduced by large differences in region of interest size while preserving anatomical interpretability. Given that co-pathology and resilience factors might not strictly follow region of interest boundaries, the super-point approach was designed to provide a less biased, finer-grained and more evenly distributed representation of MTL structure.

Second, we used clinically accessible plasma ptau_217_ as a measurement for T. Although studies have shown that ptau_217_ is strongly correlated with tau PET, it also reflects amyloid burden and does not provide spatially specific information. More granular mismatch of local tau with regions within the MTL might enhance specific patterns associated with MTL co-pathologies. However, the spatial constraints of tau PET would probably limit the yield on more precise spatial mismatch. Importantly, the approach taken here might overestimate expected MTL atrophy in atypical or cortical variants of AD, in which global tau load might be less linked to that in the MTL. One might imagine that in such cases, these individuals would be ‘misclassified’ as resilient.

Third, although the vulnerable group exhibited imaging and clinical features suggestive of LATE-NC involvement, we lack pathological confirmation. Future post-mortem studies are needed to validate this association.

Fourth, the primary T-N mismatch modelling was based on the ADNI cohort, which might not reflect the full spectrum of clinical populations. ADNI participants are generally less diverse in terms of race, ethnicity and disease severity compared with real-world patients. In the included datasets, 89% of participants in ADNI and 87% in the Penn ADRC identified as white. As such, broader validation across more heterogeneous cohorts is necessary to ensure the robustness and clinical applicability of this approach for future work.

## Conclusion

In conclusion, T-N mismatch within the MTL using MRI and plasma biomarkers revealed groups with varying vulnerability/resilience, with the vulnerable group displaying patterns of atrophy and cognition suggestive of LATE-NC. This approach provides important prognostic information and is a potentially cost-effective method for stratifying individuals for therapeutic interventions. Such stratification will probably be important in current and future interventions, given the heterogeneity of AD, which requires a precision medicine-based approach. To support broader use, we have released a public software package (https://github.com/xueying-lyu/mismatch) implementing user-customized mismatch clustering and individual-level T-N phenotypic assignment as developed in this study.

## Supplementary Material

awag105_Supplementary_Data

## Data Availability

Data from ADNI used in this study are publicly available at adni.loni.usc.edu. Data from the Penn ADRC and ATM cohorts are available from the corresponding author upon reasonable request.
