# Supplementary material for: Medial temporal lobe tau–neurodegeneration mismatch from structural imaging and plasma biomarkers
Source: Brain. 2026 Apr 28;149(11):3890–904. doi: 10.1093/brain/awag105 (PMC13480308; doi:10.1093/brain/awag105)
Supplement: Supplementary file 1 [file awag105_supplementary_data.pdf]

Supplementary Methods

Participants

Data used in the preparation of this article were obtained from the Alzheimer's Disease Neuroimaging Initiative (ADNI) database (adni.loni.usc.edu). The ADNI was launched in 2003 as a public-private partnership, led by Principal Investigator Michael W. Weiner, MD. The primary goal of ADNI has been to test whether serial magnetic resonance imaging (MRI), positron emission tomography (PET), other biological markers, and clinical and neuropsychological assessment can be combined to measure the progression of mild cognitive impairment (MCI) and early Alzheimer's disease (AD).

Participants were selected from ADNI based on the following criteria: (1) amyloid-positive (A+) status that were either cognitively unimpaired, mild cognitive impairment (MCI) or dementia, (2) availability of a pair of plasma $ptau_{217}$ and T1-weighted MRI scans acquired within one year of each other, (3) successful completion of T1-weighted MRI image processing, and (4) passing quality control (QC) for image processing.

Image acquisition

The image acquisition methods for ADNI have been previously described[1,2]. Briefly, The T1-weighted MRI scans were acquired with a spatial resolution of $1.0 \times 1.0 \times 1.0$ mm^3 or $1.0x1.0x1.2mm^3$.

Structural MRI data for the Penn ADRC study were collected on a Siemens Prisma 3 tesla (T) scanner using a 3D T1-weighted MPRAGE sequence, featuring the following parameters: TR = 2400 ms, TE = 2.24 ms, TI = 1060 ms, flip angle = 8°, field of view = $256 \times 240 \times 167$ mm^3, and voxel dimensions of $0.8 \times 0.8 \times 0.8$ mm^3.

For the ATM program, T1-weighted structural MRI scans were acquired using Siemens 1.5T (MAGNETOM Sola, Avanto) and 3T scanners (Prisma, Skyra, Vida). Most scans employed a 3D MPRAGE or similar sequence (TR ≈ 2300 ms, TE ≈ 2.9 ms, TI ≈ 900 ms, flip angle ≈ 8–9°, voxel size ≈ 0.8x0.8x0.8 mm^3 or 1.0x1.0x1.0 mm^3).

Plasma $ptau_{217}$

The tau positivity threshold was determined in a previously described cohort[3] with both Tau-PET and plasma measures available[3] to identify the $ptau_{217}$ value with the maximal Youden index for identifying individuals exceeding a Tau-PET threshold > 97.5th percentile of cognitively unimpaired, amyloid negative (A-) individuals.

Imaging signature of LATE-NC

A prior postmortem study[4] of MTL comorbidities demonstrated that the thickness ratio between the entorhinal cortex and parahippocampal cortex (ERC/PHC) was reduced in AD patients with TDP-43 co-pathology. Based on this, we used the ERC/PHC ratio as an imaging surrogate for LATE-NC and compared this metric across the T-N groups in this work.

Sampled Iterative Local Approximation (SILA)

To anchor longitudinal analyses within the disease time course, we used SILA[5] to estimate individuals' Tau onset age. Briefly, SILA uses short-term longitudinal follow-up data to approximate a single trajectory for change in a given biomarker over disease duration. This then allows for estimation of the age at which a biomarker becomes abnormal from any single timepoint. For our study, we combined data from 397 A+ individuals in the ADNI and Penn ADRC cohorts with longitudinal $ptau_{217}$ available to generate a p-tau_{217} trajectory with a

threshold of 0.3175 for tau positivity (as described above). We then used this trajectory to estimate tau onset age for each participant in our dataset.

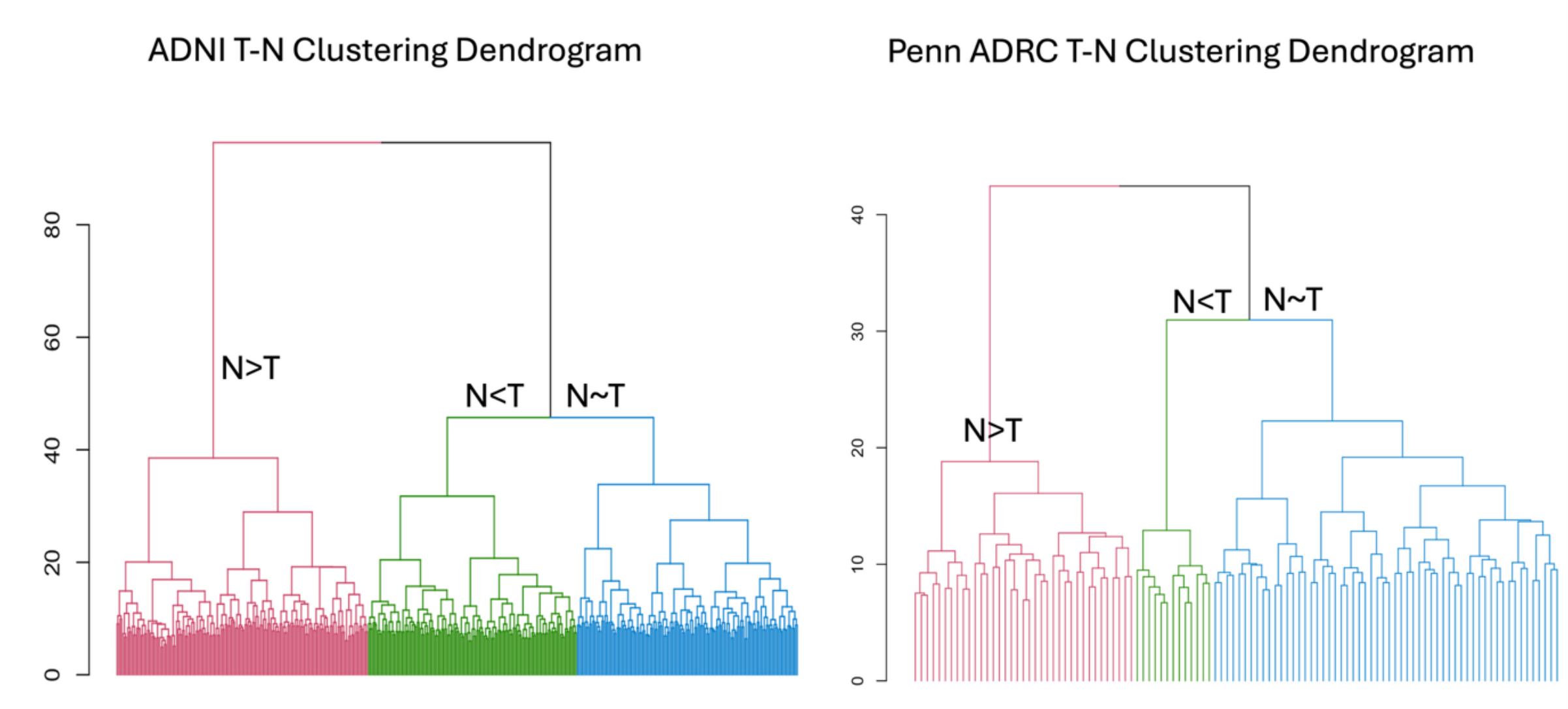

Figure S1. Hierarchical clustering dendrogram for ADNI T-N mismatch clustering (A) and Penn ADRC replication T-N mismatch clustering (B).

Table S1. Clinical characteristics of A+ subjects from ADNI, Penn ADRC and ATM

Cohort	Age	Sex (Female/ Male)	$ptau_{217}$	Diagnosis (CU/ MCI/ Dementia/ unknown)	CDRSB
ADNI (447)	76.1 (7.7)	220/227	0.475 (0.38)	163/159/125/0	2.35 (7.7)
Penn ADRC (n=108)	74.6 (6.6)	61/47	0.519 (0.41)	39/36/32/1	1.84 (2.3)
ATM (N=50)	72.5 (8.0)	27/23	0.66 (0.44)	1/29/20/0	

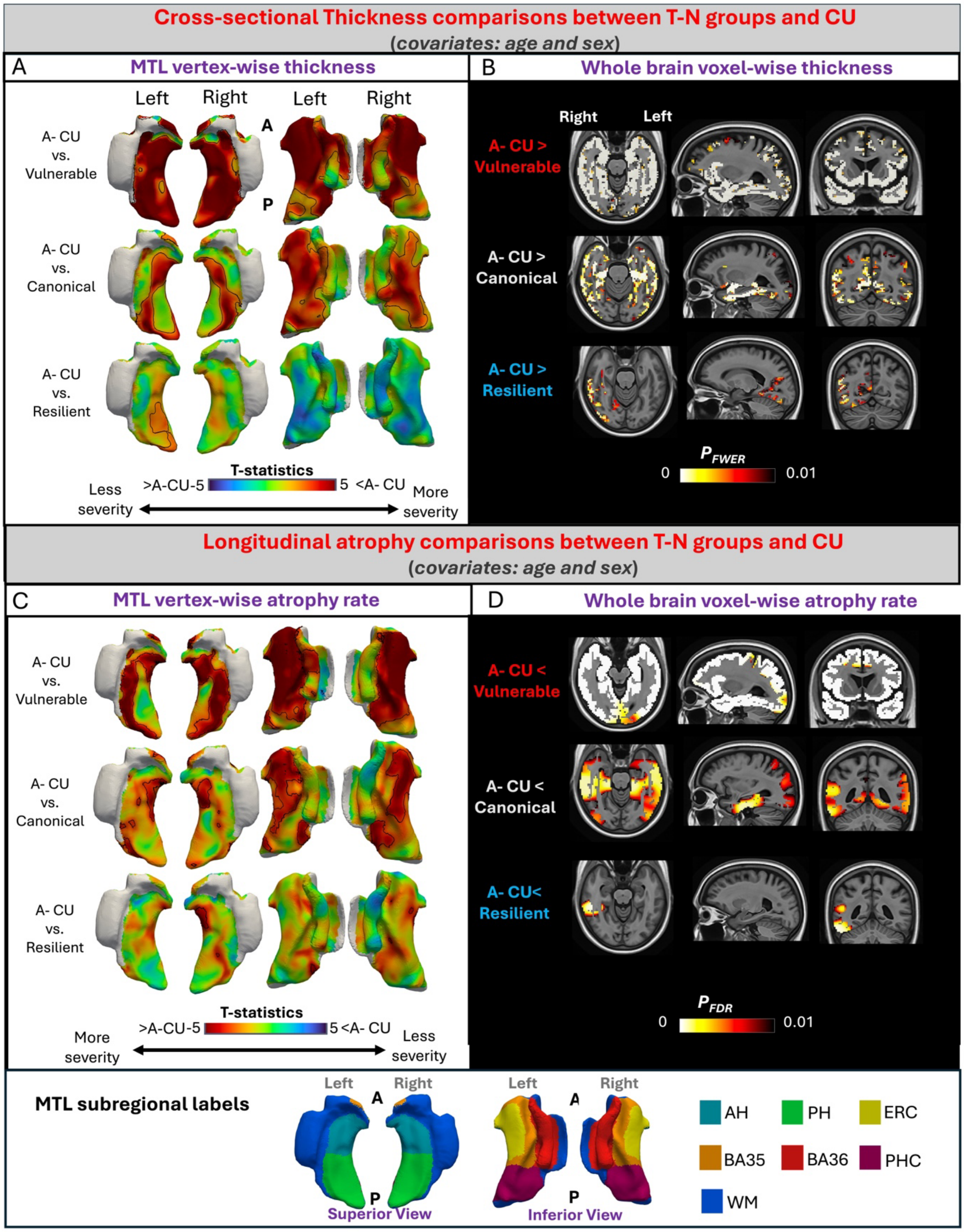

Figure S2. (A) T-statistics for point-wise cortical thickness comparisons between controls (CU) and each T–N group (vulnerable, canonical, resilient), adjusted for age and sex. Positive values indicate reduced thickness in T–N groups. Black outlines denote significant regions ($P_{FWER} < 0.01$). **(B)** Voxel-wise cortical thickness comparisons across the whole brain between controls and each T–N group ($P_{FWER} < 0.01$), adjusted for age and sex. **(C)** T-statistics for longitudinal point-wise atrophy comparisons using linear mixed-effects models, adjusted for age and sex. Negative values indicate greater atrophy in T–N groups ($P_{FDR} < 0.01$). **(D)** Voxel-wise longitudinal cortical thinning differences between

controls and each T–N group ($P_{FDR} < 0.01$), adjusted for age and sex. **Abbreviations:** **MTL**=Medial temporal lobe, **A- CU**= Amyloid-negative cognitively unimpaired, **Can**=Canonical, **AH**= Anterior hippocampus, **PH**=Posterior hippocampus, **ERC**=Entorhinal cortex, **BA35**= Brodmann area 35, **BA36**= Brodmann area 36, **PHC**=Parahippocampal cortex, **WM**=White matter, **T**=Tau, **N**=Neurodegeneration.

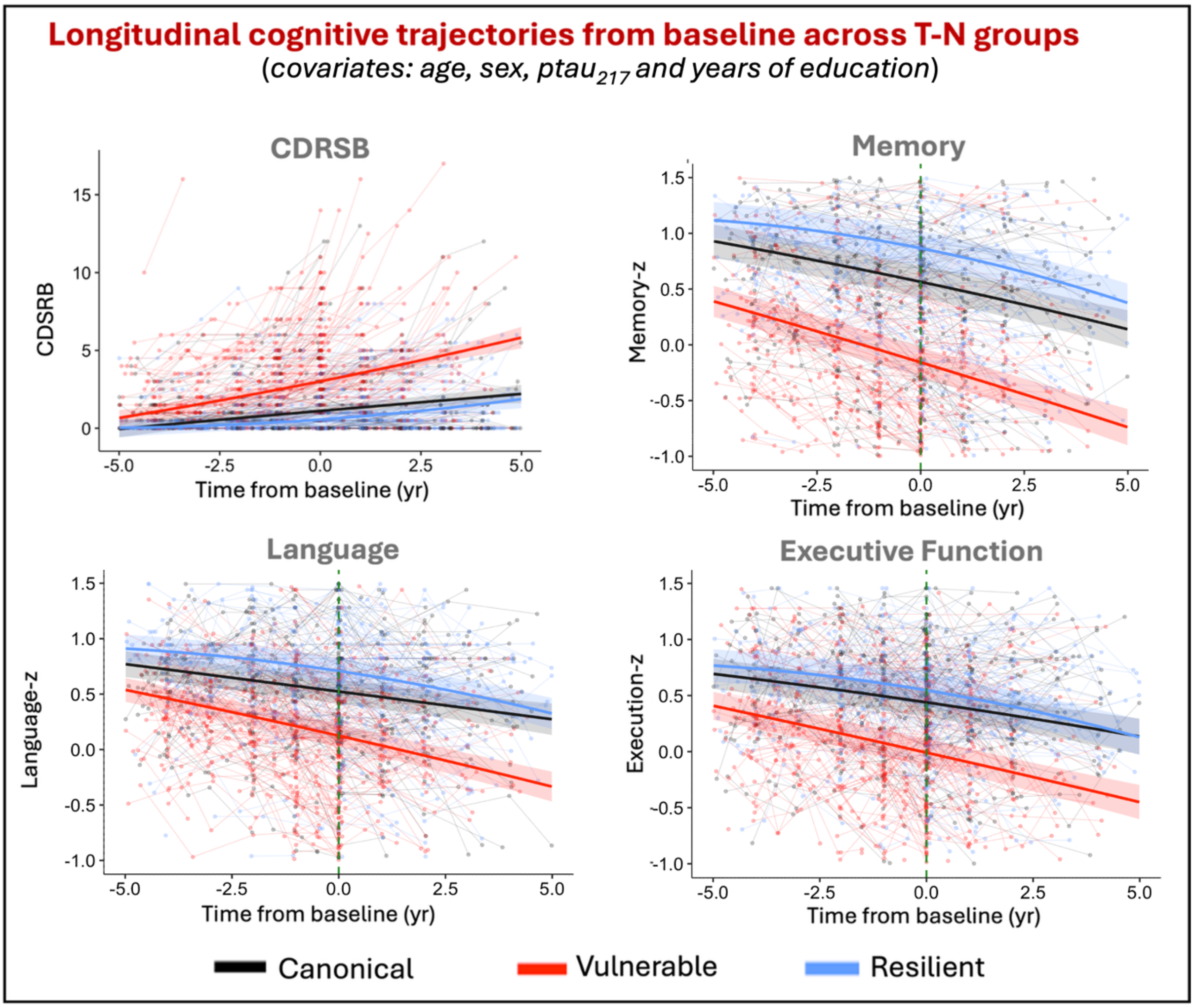

Figure S3. Longitudinal cognitive trajectories from baseline across CDRSB, memory, language and executive function z-scores between T-N groups. Shaded ribbons indicate 95% confidence intervals for model-predicted trajectories, while semi-transparent spaghetti lines represent individual individual-level cognitive trajectories over time.

Table S2. Comparisons of clinical features between T-N groups from Penn ADRC cohort (n=108). Overall group effects (p-values indicated in the bottom row) were tested using the Kruskal-Wallis test for categorical variables and linear regression for continuous variables. The comparison of $ptau_{217}$ was controlled by age and sex. The covariates for CDRSB comparison included age, sex and $ptau_{217}$. The covariates for imaging volumes or feature comparisons included age, $ptau_{217}$, sex and ICV. Only significant pairwise comparisons between T-N groups and canonical group are marked in the table alongside the corresponding value. P-values were adjusted by Bonferroni multiple comparison correction (*P<0.05, **P<0.01, ***P<0.001). Abbreviations: AH=Anterior Hippocampus, PH=Posterior Hippocampus, ERC=Entorhinal Cortex, PHC= Parahippocampal Cortex

	**Basic Characteristics** *Covariates for $ptau_{217}$ comparison: age, and sex*				**Imaging Features** *Covariates: $ptau_{217}$, age, sex and ICV (ICV not included for ERC/PHC)*				**Cognition** *Covariates: $ptau_{217}$, age, sex and education*
**Description**	**Age (yrs)**	**Sex (F/M)**	**$ptau_{217}$**	**Diagnosis (CU/ MCI/ Dem/ unknown)**	**AH Vol (mm^3)**	**PH Vol (mm^3)**	**ERC/ PHC Ratio**	**Amyg Vol (mm^3)**	**CDRSB**
Group1 Vulnerable (N>T) (n=13)	78.3 (6.1) *	5/8	0.521 (0.46)	0/ 6/ 7	1345 (248) **	1159 (156) ***	0.868 (0.098) ***	918 (149) ***	3.04 (3.5)
Group2 Canonical (N~T) (n=58)	75.1 (6.5)	34/24	0.474 (0.27)	23/ 16/ 18/ 1	1613 (273)	1525 (192)	1.01 (0.099)	1109 (129)	1.94 (2.4)
Group3 Resilient (N<T) (n=37)	71.7 (7.5) *	22/15	0.53 (0.37)	16/ 14/ 7	1817 (226) ***	1621 (220)	1.04 (0.089)	1297 (113) ***	1.29 (1.6)
Group Diff.	*P< 0.001*	*P= 0.38*	*P= 0.912*	*P= 0.011*	*P< 0.001*	*P< 0.001*	*P< 0.001*	*P<0.001*	*P=0.015*

Table S3. Comparisons of clinical features between Assigned T-N groups from ATM cohort (n=50). Overall group effects (p-values indicated in the bottom row) were shown below. The comparison of $ptau_{217}$ was controlled by age and sex. The covariates for CDRSB comparison included age, sex and pTau217. The covariates for imaging volumes or feature comparisons included age, $ptau_{217}$, sex and ICV. Only significant pairwise comparisons between T-N groups and canonical group are marked in the table alongside the corresponding value. P-values were adjusted by Bonferroni multiple comparison correction (*P<0.05, **P<0.01, ***P<0.001). Abbreviations: AH=Anterior Hippocampus, PH=Posterior Hippocampus, ERC=Entorhinal Cortex, PHC= Parahippocampal Cortex

**Basic Characteristics** *Covariates for $ptau_{217}$ comparison: age, and sex*				**Imaging Features** *Covariates: $ptau_{217}$, age, sex and ICV (ICV not included for ERC/PHC)*				
**Description**	**Age (yrs)**	**Sex (F/M)**	**$ptau_{217}$**	**Amygdala Vol (mm^3)**	**AH Vol (mm^3)**	**PH Vol (mm^3)**	**Hippocampal Vol (mm^3)**	**ERC/PHC**
Group1 Vulnerable (N>T) (n=18)	76.0 (6.0)	10/8	0.495 (0.2) *	989 (159) ***	1409 (197) ***	1347 (212) **	2757 (344) ***	0.971 (0.094) *
Group2 Canonical (N~T) (n=22)	72.8 (7.8)	12/10	0.617 (0.4)	1142 (131)	1661 (249)	1461 (178)	3122 (385)	1.04 (0.076)
Group3 Resilient (N<T) (n=10)	65.4 (7.4) *	5/5	1.08 (0.6)	1240 (138)	1831 (354)	1532 (189)	3363 (463) *	1.04 (0.082)
Group Diff.	*P<0.01*	*P=0.96*	*P<0.01*	*P<0.001*	*P<0.001*	*P<0.05*	*P<0.001*	*P<0.05*

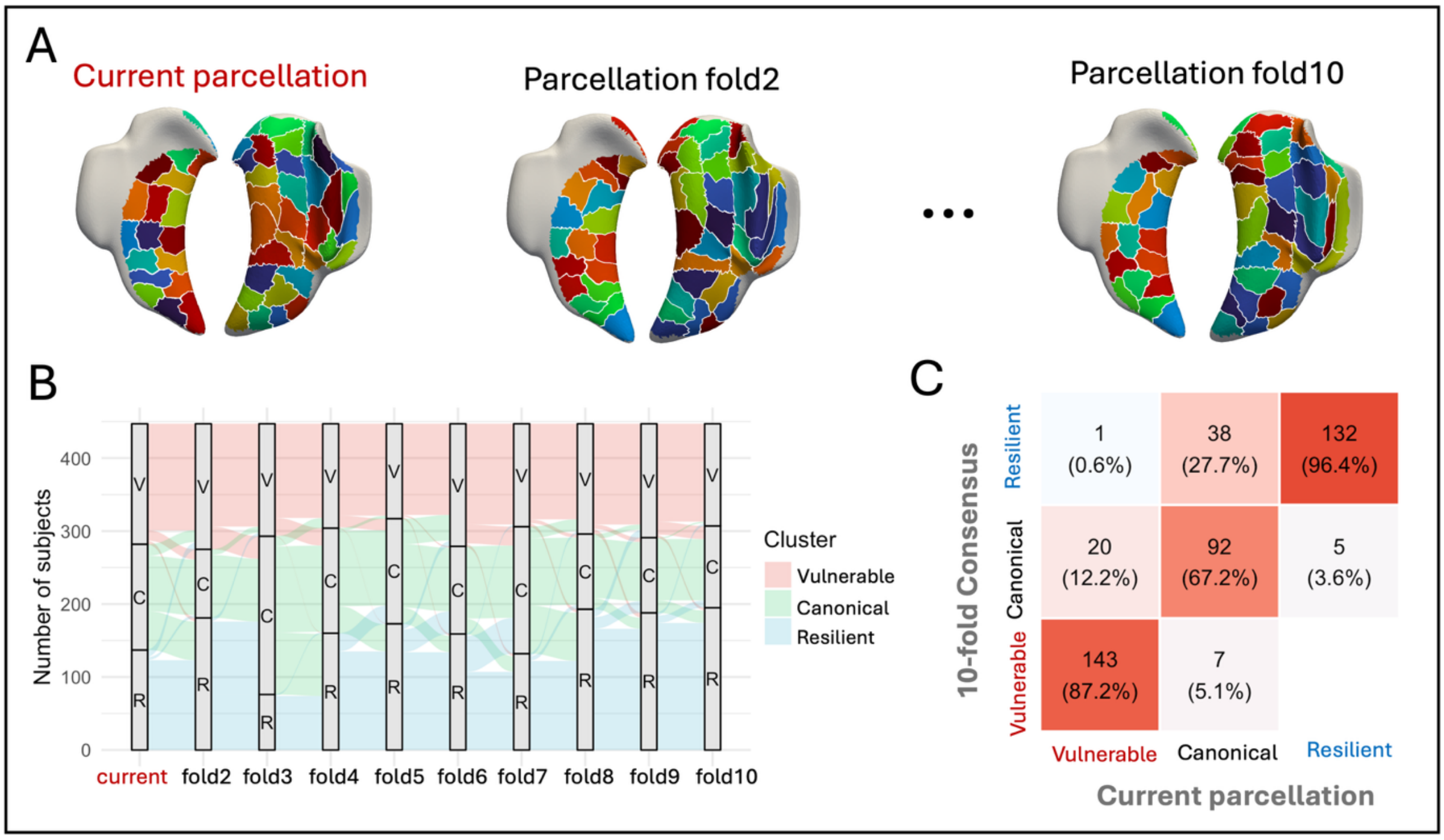

Figure S4. **(A)** Illustration of the original super-point parcellation along with representative super-point parcellations generated across the 10 cross-validation folds. **(B)** Sankey plot illustrating the consistency of T–N group membership across the 10-fold cross-validation super-point parcellations. **(C)** Confusion matrix showing the consistency of group membership between the current T–N groups and the consensus T–N groups derived by majority voting across the 10 folds. The number of patients and percentage of consistency were shown in the matrix.

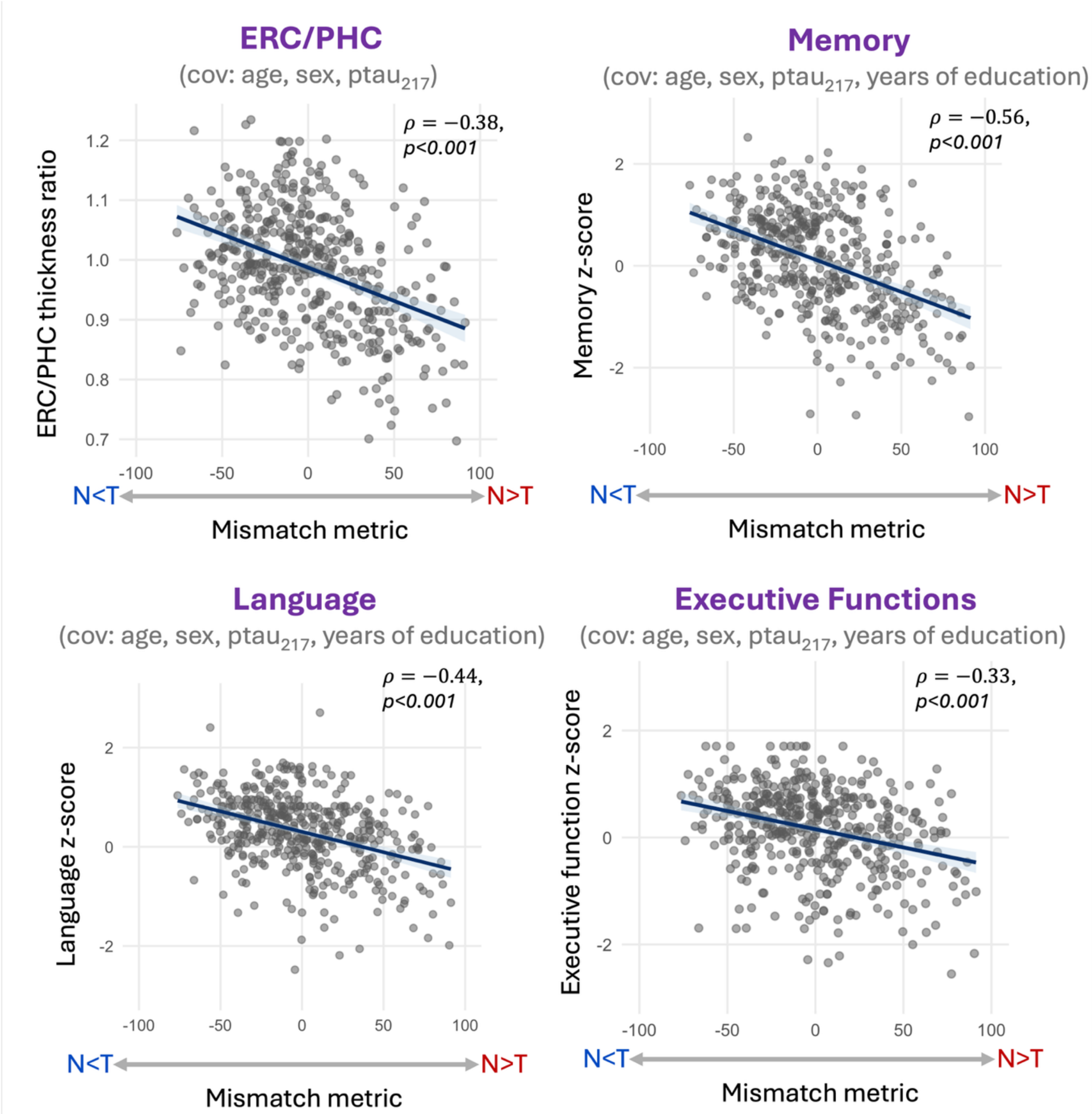

Figure S5. Associations between T-N mismatch metric and ERC/PHC thickness ratio, memory, language and executive function. The corresponding covariates were marked in the figure.

References

1. Das SR, Lyu X, Duong MT, et al. Tau-Atrophy Variability Reveals Phenotypic Heterogeneity in Alzheimer's Disease. *Ann Neurol*. 2021;90(5):751-762. doi:10.1002/ana.26233

2. Lyu X, Duong MT, Xie L, et al. Tau-neurodegeneration mismatch reveals vulnerability and resilience to comorbidities in Alzheimer's continuum. *Alzheimers Dement*. 2024;20(3):1586-1600. doi:10.1002/alz.13559

3. Brown CA, Das SR, Cousins KAQ, et al. Tau burden is best captured by magnitude and extent: Tau-MaX as a measure of global tau. *Alzheimers Dement*. 2025;21(7):e70346. doi:10.1002/alz.70346

4. de Flores R, Wisse LEM, Das SR, et al. Contribution of mixed pathology to medial temporal lobe atrophy in Alzheimer's disease. *Alzheimers Dement J Alzheimers Assoc*. 2020;16(6):843-852. doi:10.1002/alz.12079

5. Betthauser TJ, Bilgel M, Koscik RL, et al. Multi-method investigation of factors influencing amyloid onset and impairment in three cohorts. *Brain*. 2022;145(11):4065-4079. doi:10.1093/brain/awac213
